# Supplementary material for: Secondary vectors of Zika Virus, a systematic review of laboratory vector competence studies
Source: PLoS Negl Trop Dis. 2023 Aug 31;17(8):e0011591. doi: 10.1371/journal.pntd.0011591 (PMC10499269; doi:10.1371/journal.pntd.0011591)
Supplement: S1 Table — (DOCX) [file pntd.0011591.s001.docx]

**Table S1.** Grading tool developed for quality assessment of VC studies using Reporting of Observational Studies in Epidemiology (STROBE) and Strengthening of the Reporting of Molecular Epidemiology for Infectious Diseases (STROME-ID) criteria.

|  | # | STROBE/STROME-ID | | OUR APPLICATION (Maximum Points) |
| --- | --- | --- | --- | --- |
| **Title and abstract** |  |  | |  |
| Introduction  (max pts=2) | 1 | STROBE: Indicate the study’s design with a commonly used term in the title or the abstract; Provide in the abstract an informative and balanced summary of what was done and what was found | | (a) Used term VC in title (1)  (b) applicable (1) |
| Background Rationale  (max pts=1) | 2 | STROBE: Explain the scientific background and rationale for the investigation being reported; STROME-ID: Provide background information about the pathogen population and the distribution of pathogen strains within the host population at risk | | 1. applicable (1) |
| Objectives  (max pts=2) | 3 | STROBE: State objectives, including any prespecified hypotheses | | 1. State objectives clearly, especially if transmission is being evaluated (1). 2. Are the stated study objectives consistent with the methods employed (1). |
| **Methods** |  |  | |  |
| Study design  (max pts=12) | 4 | STROBE: Present key elements of study design early in the paper; STROME-ID: Describe any methods used to detect multiple-strain infections and measure their effect on the study findings | | 1. applicable (1) 2. clear indicate mosquito species and virus being tested (1) 3. appropriately reference virus strains used (1) 4. State if virus is low or high passage (1) 5. Use low passage virus: < 20 passages (1) 6. Used multiple virus strains: 2 or 3 strains (1); > 3 strains (2). 7. Clear description of mosquitoes used (source is recent field or colony) (1) 8. Generations indicated: if F_1-4_ (2), F_5-10_ (1), > F_10_ (0); not stated (0) 9. Inclusion of an appropriate measurement of transmission (2) |
| Molecular Terminology  (max pts =1) | 5 | STROME-ID: Define or cite definitions for key molecular terms used in study | | 1. Clearly describe virus strains used (note reference) (description of high or low passage), Mosquito strains used number of generations in the laboratory) (1) |
| Laboratory Methodology  (max pts =1) | 6 | STROME-ID: Describe sample collection and laboratory methods, including any methos used to minimize and measure cross-contamination, and give criteria used to interpret strain classification | | 1. Assays used to measure clearly described. Able to distinguish if an infectious assay was used to detect live virus compared to detection of RNA by PCR (1). |
| Setting (max pts =2) | 7 | STROBE: Describe the setting locations, and relevant dates. STROME-ID: State timeframe of study | | 1. Use local mosquito vectors (1) 2. Use mosquito strains from endemic/epidemic locations (1) |
| Variables  (max pts =2) | 8 | STROBE: Clearly define all outcomes. Give diagnostic criteria, if applicable. | | 1. clear definition of infection, dissemination, transmission used in analysis (2); partial (1), no definition or incorrect definition (0) |
| Data (max pts =1) sources/measurement | 9 | STROBE: For each variable of interest give sources of data and details of methods of assessment. | | (a) Diagnostic criteria: Use of infectious virus assay or animal model (1); PCR only (0) |
| Bias (max pts =1) | 10 | STROME-ID: Describe any efforts made to address discover or ascertainment bias | | 1. Use of Ae. aegypti and/or albopictus comparator (1) |
| Study size  (max pts =3) | 11 | STROBE: Explain how the study size was arrived at. STROME-ID: Describe any unique restrictions placed on the study sample size | | 1. n per group indicated (1) 2. if n per group > 20 (1) 3. Inclusion of replicates (1) |
| Statistical Methods  (max pts =1) | 12 | STROBE: Describe statistical methods | | 1. Clear description of statistical methods used for analysis (1) |
| **RESULTS** |  |  |  |  |
| Other analyses  (max pts =5) | 13 | STROBE: Report other analyses done | | 1. Infection rates reported (1) 2. dissemination rates reported (1) 3. transmission rates reported (2) 4. variability reported (e.g.., 95% CI) (1) |
| **Discussion** |  |  |  |  |
| Key results  (max pts =1) | 14 | STROBE: Summaries key results with reference to study objectives | | 1. summary of key results with reference to study objectives (1) |
| Limitations  (max pts =1) | 15 | STROBE: Discuss limitations | | 1. Here if missing some of the best practices above should be in the discussion (1) |
| Interpretation  (max pts =1) | 16 | STROBE: Give cautious overall interpretation of results considering objectives, limitations, multiplicity of analyses, results from similar studies and other relevant evidence | | 1. (1) |
| Generalizability  (max pts =1) | 17 | STROBE: Discuss the generalizability (external validity) of the study results. | | 1. (1) |
