# Supplementary material for: Secondary vectors of Zika Virus, a systematic review of laboratory vector competence studies
Source: PLoS Negl Trop Dis. 2023 Aug 31;17(8):e0011591. doi: 10.1371/journal.pntd.0011591 (PMC10499269; doi:10.1371/journal.pntd.0011591)
Supplement: Attachment — Submitted filename: Bisia_et_al_review.docx [file pntd.0011591.s003.docx]

Review of the manuscript “Secondary vectors of Zika Virus, a systematic review of laboratory vector competence studies ” by Bisia *et al.*

In the manuscript entitled “Secondary vectors of Zika Virus, a systematic review of laboratory vector competence studies”, Bisia *et al*. present the result of a systematic literature review of *Aedes* vector competence for Zika Virus (ZIKV), conducted on a myriad of scientific articles, extracted from the most relevant search engines (PubMed, Cochrane Library, LILACS, Web of Science, WHOLIS, Google Scholar).

Vector competence (VC), an evaluation of the vector capability to transmit a pathogen, provides a summary statistic for a two-fold process: i) the ability for a vector to infect itself upon feeding on contaminated blood, and ii) its subsequent ability to infect back another host (human, in the case of ZIKV). To be fully potent for that second step, viral particles in the mosquito are required to breach multiple barriers, to travel the midgut all the way to salivary glands.

The key findings of the paper are:

1) The large heterogeneity in reported outcomes and terminology, along with the difficulties this causes to integrate results into broader studies such as meta-analyses,
2) Identification of *Ae. japonicus* and *Ae. vexans* as potential vectors of interest ZIKV spread,
3) Overall poor VC for *Culex* *quinquefasciatus*.

The authors also identify critical methodological issues that may hamper the generalization of previously-published results.

**Overall, the article provides a good summary of the current state of scientific research for ZIKV vector competence, and I believe it would be of benefit to the readership of PLOS NTDs provided the author address the comments summarized below**. In particular, the introduction is carefully written and does an excellent job at detricating *vector competence* from *vectorial capacity*, two notions that are often mistakenly used. The methodology used for the systematic review is sound and the research question well phrased in the first sentence of the Methods section.

My main concern for this article revolves around the timing for publication: the literature search was stopped as of March 15^th^, 2022 (more than a year ago at the time of writing this review) and since then, at least one major publication that corresponds entirely to the “future work” authors are calling for has been published (Obadia *et al.*, <https://doi.org/10.1038/s41467-022-32234-y>).

# Major comments

**Major comment #1**

My first obvious comment arises from the “Interpretation” section of the abstract: I fully concur that future research initiatives are needed to develop vector competence laboratory experiments standardized across countries, in an effort to properly characterize VC while accounting for Gene x Environment factors. In fact, in Table 1, several of the selected publications were from teams associated with the ZIKAlliance consortium (<https://zikalliance.tghn.org/>), a Horizon 2020 European research program. The same consortium published in August 2022 a consolidated analysis of such a standardized effort, by studying 12 countries, 50 mosquito populations from 6 different species, including sampling at multiple locations in many countries. *Aedes* and *Culex* mosquitoes were among those studied, while 6 ZIKV genotypes were used for challenges. The findings are in general agreement with the present literature review regarding the concern for *Ae. japonicus* and the lack of susceptibility for *Culex*. I don’t understand why the authors stopped their literature review as of March 2022, but this study definitely addresses some of the “future research initiatives” that are called for.

Why did the author stop including articles as of March 2022 ? We are now a year later and literature has apparently evolved on that topic, which would make some statements (see just above) partially addressed.

This comment overall applies in the discussion as well (L625–627).

**Major comment #2**

The scoring system used for the “Quality assessment” is at the core of grading the 45 studies that made it into the literature review. I can only appreciate how complex building such a scoring system is, and that some decisions have to be made eventually. The discussion however lacks a section debating such decisions and the relevance of the scoring values. I note that the two scores (total score (TS) and methodology score (MS)) correspond to the total sum of items and that no weight is applied. This is partly compensated by the DELPHI-like approach where two researchers from the team scored independently each study and debated until reaching consensus. This however does not challenge the weights given to every item. Furthermore “reaching consensus” may be viewed from a mathematical aspect as a sort of average, though between-investigator variation is an important source of noise that should be considered. At minimum, some more explanations about the procedure in case of discrepancies is warranted:

- Are there specific articles that made it challenging to reach consensus consensus? If so, which ones, and how as this handled?
- Are there specific items that showed consistent variations across grading researchers? This would imply differences in interpretation and would make the scoring system difficult to rely on for that item.

L673–674 in the discussion also calls for that comment. There is no point in “rewarding” another article but rather develop a scoring system that provides quantitative insight about a paper’s methodology.

**Major comment #3**

As a follow-up comment about that scoring system, Figure 2 provides the distribution of TS and MS indicators. This figure does not read well and would benefit from better plotting in a true histogram-like manner (where the area of each rectangle would reflect on its probability). The boxplots are again hard to read (median and whiskers being hardly distinct from the background color). Putting MS and TS side by side lead the reader into thinking these two distributions are meant to be compared, though they aren’t. My suggestion would be to rethink that figure to show the overall distribution using only one panel (either combining histograms and a horizontal boxplots, or simply a histogram), possibly faceting by type of score. The aesthetics should be enhanced, too (font size, axis labelling etc.) In the corresponding section of the text (L301–309), please also indicate what the error margin relates to (“the mean score was 29.0 ± 4.2”: what is that 4.2 value?). Looking at the distributions in Figure 2A, there are evidence of bimodal distributions around lower and high end of the possible score range (e.g. 2 gaussian distributions in each score). This is in line with my previous concern for the need of debating the relevance of the retained scoring mechanism: I would rather expect one single gaussian for each score, as in its current state this points towards difficulties for reaching an “average-level study”.

**Major comment #4**

In the paragraph “Review of evidence for individual vector species other than *Ae. aegypti* and *Ae. albopictus*”, the authors identify 4 context of epidemiological relevance. I believe some of these (especially #1 and #2) would correlate with the usual definition of occupational (#1) vs. peri-domestic (#2) exposure to mosquito bites. Mentioning the context for mosquito exposure would be useful as an epidemiological descriptor for the population at-risk. Furthermore, there are 4 categories identified here though 5 are detailed in Table 5: presumably, the “Other” section was added to include the *Anopheles* species? Please modify the main text to help identify which category corresponds to which in Table 5.

**Major comment #5**

In the discussion (L591–592), the authors emphasize the need for reporting standards. A natural follow-up to that systematic review would be a meta-analysis of infection/dissemination/transmission outcomes. The authors should consider mentioning how study heterogeneity can be estimated should such an approach be taken (Cochrane’s Q, Higgins’ and Thompson’s I^2^, among others).

# Minors comments

**Minor comment #1**

The section “Time and geographical clustering of studies” is useful to summarize study contents but would be well supplemented by a visual aid so that the reader could quickly realize how well the selected studies cover the areas of interest for ZIKV. Could the author come up with a global map detailing at the continent(country)-level how many studies apply to a region and how these studies are split across the three time windows (pre/per/post ZIKV epidemic)?

**Minor comment #2**

The ”Vector competence parameters” section does not discuss the temperature used across experiments. Temperature (and, somewhat related, altitude) has been shown to positively correlate with infection/dissemination/transmission efficiency through various biotic and abiotic processes (see Bellone *et al.*, <https://doi.org/10.3389/fmicb.2020.584846>; Mercier *et al.*, <https://doi.org/10.1038/s41598-022-10977-4> though the latter does not focuses on ZIKV but other arboviruses). Surely, among the 45 studies, this important parameter was often reported and integrating it would be useful in that section. Likewise, the time since inoculation is hardly mentioned although it is often positively associated with the outcomes of interest.

**Minor comment #3**

The last paragraph of “Studies using mice” is sometimes difficult to read (esp. L452–461).

**Minor comment #4**

Table 5 is a very useful summary of all the paragraphs looking at evidence for vector competence by species. It however lacks description of how transmission efficiency (TE) is calculated. Judging by the low proportions, I’m inclined to believe this corresponds to the number of mosquitoes with viral particles in saliva divided by the total numbers of mosquitoes challenged. The TE column is also not explicit : does it correspond to the range of values in the Transmission column ? Please add detail in the legend. The “NT” abbreviation is mentioned in the legend but not used in the table (variations: ND, NT, Not tested). That table needs to be formatted slightly better.

**Minor comment #5**

L530 : This is not unexpected as increasing TE with temperature has also observed with other arboviruses, too.

**Minor comment #6**

The two paragraphs for *Ae. japonicus* and *Ae. vexans* (L547–552) appear in a very unexpected manner. Consider integrating into a single paragraph identifying vectors of potential interest esp. in settings as #3 (L469) ?

**Minor comment #7**

L558–559 : please clarify the sentence.

**Minor comment #8**

In the paragraph “Possible role of *Culex* species”, the overall consensus is that *Culex* is hardly competent to transmit ZIKV, except for the (unproven) possibility of geographically isolated species. I believe this paragraph would benefit from a stronger phrasing of that overall *Culex* poor vectorial competence (in particular L566: I do not believe that these studies overall indicate that “*Culex* species [are] able to transmit”, but rather they show a very poor vector competences overall).

**Minor comment #9**

In the discussion (L629–633), the authors highlight that science may be rushed in times of uncertainty. History has repeated itself at an even larger scale over the past few years. This is not unusual and in my opinion shows a strong mobilization. If the aim of that paragraph is to emphasize that early, rapid papers should be interpreted more cautiously than those published a few years later, this message should be made clearer.

**Minor comment #10**

L728: please detail the qualitative differences the authors are referring to.

**Minor comment #11**

L783–784: I do not understand the reluctance of authors for “small number of occurrences” (this happened earlier in the text but did not catch my attention until the phrasing I’m reporting here). First, the phrasing here is provocative and uncalled for. Second, rare events can be studied using appropriate statistical methods, the first coming to mind here being exact Binomial tests. In the case of *Culex*, how would one be able to characterize low/nil transmission rates other than reporting 0, 1, 2 mosquitoes out of more?

Similarly, L794, I respectfully disagree: 1 mosquito out of 69 is **clearly** way below the 20 infected *Aedes* out of 20 (100%) and also below the 14 (70%) that had disseminated the virus. The whole point of a title is to summarize the key findings in context. What is the point of that comment ?

**Other minor suggestions**

L195: typo in the word “competence”.

L262: typo in the word “appropriately”.

L335: de-italicize “were tested”.

L411: the verb is missing.

L418–419: a verb might be missing, or sentence should be rearranged?

L420–421: check syntax for last sentence of the paragraph.

L610–614: please rephrase.

L616–617: how are these data “misleading”? That sentence reads very critical and does not add a lot of value. I believe that no data is often better than poor/biased/unclear/… data.

L633: typo in the word “assessing”.

L656–658: please clarify what the word “strains” refers to, as it seems it’s used interchangeably for mosquito species, geographic location of mosquito sampling and ZIKV genotypes.

L698: typo in the word “assessing”.

L769–772: overlong and unintelligible sentence, please rephrase.
