# Supplementary material for: Secondary vectors of Zika Virus, a systematic review of laboratory vector competence studies
Source: PLoS Negl Trop Dis. 2023 Aug 31;17(8):e0011591. doi: 10.1371/journal.pntd.0011591 (PMC10499269; doi:10.1371/journal.pntd.0011591)
Supplement: Attachment — Submitted filename: ReviewerResponse8June2023.docx [file pntd.0011591.s004.docx]

General Comments for Reviewers and Editors

- We would like to thank each of the reviewers who provided well considered comments, suggestions, and reviews. Each took their role as reviewers very seriously and provided commentaries that have improved the manuscript considerably. We also are encouraged that reviewers were supportive of our study objectives, including our efforts to illustrate that within the vector competence (VC) literature, “the methodology/discipline suffers from a pervasive lack of unity with respect to methodologies, definitions and other critical parameters (Reviewer #2)” in addition to our primary objective of identifying potential secondary vectors for ZIKV.
- Please note our responses are in blue text, with text directly from the manuscript in Arial Font.
- Major changes and modifications
  - Standardization and definitions (Reviewer #1 comments #9 and 17 are largely addressed here). We have taken Reviewer #1’s request in earnest and now express all titer data in the format ## log_10_ PFU/ml or TCID_50_/ml throughout the manuscript. We have either corrected and searched for all grammatical and syntax errors pointed out by all three reviewers. As part of this standardization, we now present all infection, dissemination, and transmission parameters using the terminology proposed by Yifei Wu et. al. 2022. Thus, we have added a paragraph describing the typical steps in a vector competence (VC) experiment to the introduction (see Lines 124-133). Then we describe the concept of stepwise and cumulative “rates” and define them (Lines 133-138). We address this issue more assertively throughout the manuscript and in results section, under vector competence parameters (Lines 446-449, **460-471**). The lines in bold directly address Reviewer #1’s deep concern about the fact that there are multiple definitions for the terms dissemination rate (DR) and transmission rate (TR) used in the literature. We would like to avoid placing blame or any commentary on how this situation evolved or why, but strongly acknowledge that it is an important problem. Finally, we further emphasize this issue in the discussion (Lines 806-821, 823-834).
  - We have made considerable modification to Tables 1 (Lines 317-329) and 5 (Lines 527-547), as well as changes to Table 3 (Lines 434-439).
    - Table 1. Third column is now labeled Mosquitos/Virus/Temperature. That includes virus strain and doses used for all experiments (rather than presented in the results column), and the environmental conditions of the experiments (with temperature as a minimum), requested by Reviewer #3. Finally, we focus on highlighting the raw data necessary to calculate transmission parameters, rather than summarizing findings presented by the individual authors.
    - Table 3. The primary change here, is in the Genebank No. column. We report only accession numbers reported in the original article, providing the citation. We emphasize that we went back and checked all the information in this table carefully. Finally, at the request of Reviewer #2, we clearly state that no recombinant strains were utilized in the reviewed studies.
    - Table 5. This table has changed significantly using some important suggestions from Reviewer #1 (standardization and direct comparison of stepwise/cumulative rates to highlight the cumulative transmission rate, the most relevant for deciding if a vector is a potential vector or not) and #3 (inclusion of experiments where the effect of temperature was evaluated). Rather than providing ranges reported by authors, we used the raw data to calculate parameter values for epidemiologically relevant 14 dpi (unless otherwise indicated). This allows readers to make head-to-head comparisons of both stepwise and cumulative transmission parameters. Thus, we used raw data to calculate rates rather than presenting rates selectively presented in the original article.
  - Discussion of the role of temperature on VC (Reviewer #3’s suggestion). The inclusion of temperature and other environmental conditions, for all reviewed articles, are included in table 1 in the third column. We have added a paragraph to the results section: **Role of Temperature (**Lines 643-649) and we modified Table 3, to show temperature results for 3 species.
- March 15, 2022, finalized search date (major issue for Reviewer #3). First, we acknowledge that the article by Reviewer #3 published in August 2022 is exactly the kind of multi-country effort we proposed in the original conclusion of the abstract and in our discussion. Therefore, we have removed the request for this type of study from our abstract, and now highlight this article in our discussion. As part of a systematic review, authors need to establish a final search date and stick to it. One the search is stopped, we initiated the retrieval, examination, and analysis of the included articles. The duration of the process was lengthy due to the vast quantity of papers found. Specifically in total 45 papers were selected that necessitated information extraction and the quality assessment tool that demanded meticulous attention to detail. Consequently, following this process takes time, and you need to respect the search dates you set. Nevertheless, this new information is now acknowledged and used to demonstrate best practice for future VC studies (Lines 756-765, 937-941).

Reviewer's Responses to Questions

Reviewer #1: No, please see comments 9 and 17 in my Summary and General Comments

Response: Please see General responses above and our responses to these concerns below.

Reviewer #2: -Yes

-Yes

-Yes

-N/A

-N/A

-No

Reviewer #3: See the attached review report

--------------------

Reviewer #1: I don't think that Figure 2 adds any value to the manuscript and should be deleted. **DONE, however we have added a new figure 2 suggested by Reviewer #3.**

Reviewer #2: -Yes

-Yes

-Yes

Reviewer #3: See the attached review report

--------------------

Conclusions

Reviewer #1: No, again, please see comments 9 and 17 in my Summary and General Comments

Reviewer #2: -Yes

-Yes

-Yes

-Yes

Reviewer #3: See the attached review report

--------------------

Reviewer #1: There are numerous minor editorial errors in capitalization, spelling, abbreviations, etc. Please see my Summary and General Comments

Response: We appreciated the attention to detail and have made all suggested editorial changes.

Reviewer #2: Minor Revision

Reviewer #3: See the attached review report

--------------------

Summary and General Comments

Reviewer #1: General Comment: Zika virus (ZIKV) created a major panic back in 2016-2018 when it caused millions of cases of disease in the Americas. As with the other anthroponotic arboviruses, e.g., yellow fever, dengue, and chikungunya viruses, Ae. aegypti is the principal vector. Yes, Ae. albopictus has been involved in a few outbreaks, but see comment 1 below. Because these anthroponotic viruses require the same mosquito to take two separate blood meals on a human, only anthropophilic mosquitoes that preferentially feed on humans are likely to be involved in the transmission of these viruses. This should be emphasized more in the paper. My biggest concern, as mentioned in comments 9 and 17, is that because authors use completely different definitions of a transmission rate, simply reporting the rate that these authors claimed is very misleading and may make an essentially incompetent mosquito appear highly efficient.

*Response:* We agree completely with Reviewer #1’s two broad concerns. First, and most important (comments 9 and 17) is the issue of what “rate” is reported in each article and their comparability. Please see our responses above, we have made a significant effort to standardize our reporting in Tables 1 and 5 as well as throughout the manuscript. Please also see how we have addressed the issue of definitions described under general comments.

I understand that for most of the authors, English is a second language, but there were a lot of minor inconsistencies. Editors, reviewers, and readers can’t see how the study was done, but if the writing is careless (see numerous comments below), they are less likely to believe that the authors were careful in how they did their study. **Noted.**

Specific Comments:

1. Line 46 and numerous comments throughout the entire manuscript. Yes, Ae. albopictus can readily transmit ZIKV and yes, Ae. albopictus readily feeds on humans. However, how important is Ae. albopictus in the transmission of ZIKV, chikungunya virus, or dengue virus? After their introduction into the Americas beginning in 2013, about 10,000 cases of chikungunya and Zika were diagnosed and reported in the U.S. (given the inefficiency in diagnosis and reporting, this was a severe underreporting of cases). Despite many, many reported imported cases of chikungunya and Zika in the southeastern U.S., where Ae. albopictus is one of the principal pest mosquitoes, not one case of locally transmitted chikungunya or Zika was reported from any location that did not also have a significant Ae. aegypti population. Because they are not as anthropophilic as Ae. aegypti, a single Ae. albopictus is not likely to take multiple blood meals from a human, and Ae. albopictus are not as likely to be involved in significant transmission of anthroponotic pathogens such as dengue, chikungunya, Zika, and yellow fever as Ae. aegypti. Note, in the outbreak on the islands around the Indian Ocean, there were very few other animals present to feed on, so the Ae. albopictus did take multiple blood meals from humans, and the same thing happened in a few locations in Italy.

*Response:* We agree, *Ae. aegypti* is a much better vector than *Ae. albopictus* and the reason is that all the ecological and behavior components of vector capacity help *Ae. aegypti* overcome better laboratory vector competence parameters in *Ae. albopictus* (shown nicely classic manuscript by Lambrechts, Gubler, and Scott). There are multiple publications comparing *Ae. aegypti* and *Ae. albopictus* vector competence, also a topic for a systematic review, but not under this study. The scope of our review, however, are any secondary vectors other than *Ae. albopictus.* To address this issue, we have made the following changes in the manuscript.

*Abstract: (Ln 44-46) now reads.*

*“Aedes aegypti* and *Ae. albopictus* are now well established primary and secondary ZIKV vectors, respectively. Consensus about identification and importance of other secondary ZIKV vectors remain.”

*Introduction: (Ln 95-96): removed the word primary.*

“not only about vector competence (VC) for “known” vectors *Aedes aegypti* and *Aedes albopictus*, but also on potential “unknown” secondary vectors.”

2. Lines 64-66: Why are the use of murine models and infectious virus assays critical gaps? To me, it is critical that the studies use infectious virus assays as qRT-PCR may detect some lingering, non-infectious pieces of RNA remaining from the blood meal in an uninfected mosquito resulting in it inappropriately being called positive. Similarly, the use of artificial blood meals often produce lower infection rates than when mosquitoes ingest the same virus titer from a viremic host. Therefore, it is not immunocompromised murine models that are a critical gap, but rather the lack of use of these models.

*Response: In principle we agree*: the use of murine models as well as direct feeds on viremic persons probably provide more realistic measures of vector competence, but we don’t see many head-to-head comparisons in the literature to make this point. We also agree that the use of infectious assays is critical, a main message of the current review. However, papers continue to be published (and submitted) that do not follow this criterion, and we argue that their quality is significantly lower than articles that do. Therefore, there is a need for a serious discussion if using an infectious assay should represent a minimum standard for a VC experiment, e.g. answer the following question -- Do articles/studies using only detection of viral RNA by PCR merit publication? It is logistics and economic resources that clearly limit many researchers from conducting the best science with the best study designs. We added the work “issue” in addition to gap to this section of the abstract.

Lines 59-65.

“Critical issues or gaps identified included 1) inconsistent definitions of VC parameters across the literature; 2) equivalency of using different mosquito body parts to evaluate VC parameters for infection (mosquito bodies versus midguts), dissemination (heads, legs or wings versus salivary glands), and transmission (detection or virus amplification in saliva, FTA cards, transmission to neonatal mice); 3) articles that fail to use infectious virus assays to confirm the presence of live virus; 4) need for more studies using murine models with immunocompromised mice to infect mosquitoes.”

2. Lines 86-87: I found this confusing. Testing mosquito bodies for infection is MUCH more efficient than testing midguts. Similarly, testing salivary glands for virus dissemination is very inefficient compared to testing either legs or heads. Note, I have seen papers that test heads to test the presence of virus in salivary glands, but in a mosquito, the salivary glands are in the thorax, not the head.

*Response:* We agree with your comments above, but we have not seen head-to-head comparisons of the different approaches, nor have we seen the rational for using midguts/salivary glands/ovaries over bodies and head/legs described. In other words, why do some researchers choose to use dissected parts over whole parts? Logistically, we agree that bodies, heads or legs+wings, and saliva is far more straight forward. But without evidence from the literature, the current review can only suggest that this is an issue (possibly a knowledge gap as well) that deserves discussion. The role of dissecting out salivary glands may provide additional information on the ability of the virus to enter the salivary glands representing dissemination. We make this point elsewhere in the manuscript; that positive salivary glands are not equivalent to transmission. However, only two manuscripts may have made that error, although they are somewhat ambiguous and do not clearly say that positive salivary glands demonstrate transmission (it is possible they were instructed by an editor that they could not), but it can be misleading, nonetheless. We ask if dissections of mosquito parts could result in contamination, but these protocols have washing steps to address this. Solid evidence in head-to-head comparisons of these two approaches would be welcome. See Lines 851-859.

As for line 84 we changed salivary gland to legs+wings to avoid confusion.

3. Line 110-120: Very well said!

*Response:* Thank you. We also believe this addressed, to some degree your concern expressed in your first general comment.

5. Line 295: Yes, “Days post infection (DPI)” is commonly used, but shouldn’t it be “Days post exposure (DPE)” as many of the mosquitoes did NOT become infected and in some cases, none became infected, so how can it be days post INFECTION?

*Response:* Agree, but DPE was used in 2-3 of the 45 manuscripts we reviewed. We have added text to the Table 1 legend (Line 319, 3^rd^ line of the legend) acknowledging the term. We have continued to use DPI since it is the term used in a significant majority of the reviewed articles. Additionally, we have added the following text to the discussion addressing this issue.

Lines 836-840

“Finally, in the reviewed articles the number of days after mosquitoes were fed blood infected with ZIKV was reported in days post-infection (dpi) for most articles and as days post-exposure (dpe) in a few. Although less problematic than the terms dissemination and transmission rate having distinct definitions, the term dpe is more appropriate and should be adopted to standardize terminology in the vector competence literature”.

6. Line 296: What is a Transmission rate versus a Transmission efficiency? I have seen these terms misused numerous times in recent years, and they need to be defined in this paper. Because people define these terms differently, it is important that the authors first define how they are being used in this paper and then correct the citations so that the cited paper are using the same definition, i.e., if the paper states that it is using transmission efficiency, but it is using the definition of a transmission rate (as defined in this review), then the paper should be cited as using a transmission rate despite claiming to have used a transmission efficiency.

*Response:* We now clearly include definitions in the Introduction (Lines 133-141) in the Table legends and have converted discussion of rates to the stepwise and cumulative rates described by Yifei Wu et al. This was described in detail above under the general responses.

7. Line 297 (Table 1): I found this table to be very confusing. Obviously, the various “Results” were written by different people with different formats, i.e., summary first or methods first. These all need to be rewritten to be in a similar format. Either is fine, but they need to be consistent. There are also a bunch of minor errors which I will include here, rather than in the section below on Minor Comments. It is important that the writing be consistent so that the reader can easily go from one paper to the other and compare the results. Someone needs to go through ALL of these and make sure that they are consistently presented.

Response: See modifications to Table 1 in the general responses above. We thank the reviewer for all the suggestions and issues raised. We made a concerted effort to address all of these concerns and can see that it was worth the effort.

a. Reference 68 and 69: What is USUV? It is not established in the paper. I know that it is Usutu virus, but will the readers know this? Also, ZIKV is used in many of the papers. However, ZIKV was not established in the heading for this table and ALL abbreviations need to be established in figures and tables.

*Response:* Both terms now defined in the Table legend.

b. The titers of the infectious blood meal are listed in numerous different ways. For example, it is reported as “log10 PFU/ml” (most of the papers) or as “logs” (see paper 39 and others), or “3x10(5) PFU (See paper 30), or 105.4 PFU (note, the 5.4 was in a subscript) (see paper 47), or 2,530 PFU/ML (see paper 40), or merely as particles (see reference 40). Again, the authors need to be consistent. All of these “PFU” titers should be in the same format, either X.X log10 PFU/ml (note, the “10” should be a subscript) or 10x.x PFU/ml (note, the x.x should be a superscript). Minor, but reporting a titer to two decimal places is reporting data beyond the level of accuracy and should be rounded to the nearest tenth of a log (see reference 44).

*Response:* Again, see the general responses above, all these suggestions have been considered and our reporting standardized.

c. Some of the English usage is a bit confusing. For example, in paper 45, it states, ‘Aedes aegypti and Ae. polynesiensis in ZIKV spread was also suspected in addition of Ae. aegypti, supported by its ability to transmit laboratory colonies (F16 to F18) were orally infected with a French Polynesian strain) at a 7 log10 TCID50/ml and evaluated at 2, 6, 9, 14, 21 d DPI.” This, and many of the others, needs to be completely rewritten. Note, why was it “…, 21 d DPI.?”

Response: We have made extensive modifications to Table 1, focusing on appropriate metadata need to calculate VC parameters– an attempt to provide the raw numbers in Table 1 instead of rates and focus principally on the secondary species.

d. Why is “Hemotek” listed under “Mosquitoes/Virus” for reference 37? The method of virus exposure is not list for others under this column, but rather under results for some of the references.

*Response:* Removed reference to sheep blood and Hemotek.

e. Check spacing. There were numerous places where a space was left out. For example, in paper 57, “at 9dpi,”should be at “9 dpi,” (note, see above and this should be at 9 dpe). Likewise, in paper 58, “log10PFU/ml” should be “log10 PFU/ml,…”

Response: We have checked and adjusted our formatting of this table to avoid some of these issues. That being said formatting of Table 1 has been a considerable challenge, Word tends to autocorrect some items that should not be touched, and it has been an ongoing challenge to maintain our own modifications. As mentioned previously, we have used dpi, but explain it is being used interchangeably with dpe in the Table 1 legend and in lines 836-840.

f. Why are the infectious titers given in the “Mosquito/Virus” column for some papers, but in the “Results” column for other papers. You need to be consistent. Putting these critical elements in different places makes it much more difficult for the reader.

Response: We have now standardized the virus and titer information in the mosquito/virus/temperature column.

g. Also, is it “dpi” as in paper 57 and others or “DPI” as in paper 54 and others? Likewise, is it “ml” as used in most papers, “ML” as used in paper 37, or “mL” as used in paper 77?

*Response:* All changed to DPI and ml for consistency.

h. What is a “PFUe” in paper 77?

Response: plaque forming unit equivalents, but this is now expressed as PFU/ml, although the authors of this manuscript are technically correct to use this term.

i. Why in paper 77 does it have titers expressed as “log10” with only the “0” as a subscript?

Response: Corrected

8. Line 310 (Figure 2): I’m not sure what this adds to the paper. Yes, according to the criteria selected by some of the authors, some of the papers were stronger than others, but which papers received high scores and which received low scores, so that we know which papers’ results/conclusions are more believable, is already indicated in Table 1.

*Response:* We have no objection removing Figure 2 and have done so. Please note that a new Figure 2, a map suggested by Reviewer #3 has been included.

9. The difference in how the studies defined these rates is CRITICAL! For example if study A defined the Transmission Rate (TR) as the number of mosquitoes transmitting virus divided by the number of mosquitoes with a disseminated infection tested and study B defined the TR as the number of mosquitoes transmitting virus divided by the number tested (this is what is said on lines 418-420), and the actual numbers were 100 tested, 50 infected, 3 with a disseminated infection, and 2 transmitting, then in study A, the TR was 67%, yet in study B, it was 2%. How will the reader know which it really was? Again, you need to state one definition and adjust the rates so that the studies are comparing the same thing. As written, the species tested in study A would appear to be a highly effective vector, while those in study B would appear to be very poor vectors. This can be VERY misleading as if in study A, the numbers were above for mosquito species X, but in study B, the numbers were 100 tested, 50 infected, 20 with a disseminated infection, and 20 transmitting for mosquito species Y, so if study B used the definition for study B, it would report a TR of 20% for species Y, so in your paper, you would report that species X has a TR of 67%, while species Y has a TR of only 20%, despite the fact that the actual TR of species Y was 10-fold higher than species X. If you report the rates from the various studies, which this paper needs to do, you NEED to state and use a consistent definition of that rate, so if the TR is the percentage of all mosquitoes tested (which is the classical definition), then the rate for study A needs to be changed to 2%, even if they reported 67%. That allows the reader to compare the results of the various studies. If this is not done, then this paper is completely misleading and should not be published!

Response: We appreciate the reviewer’s passion on this topic, which stimulated us to review each article again from this perspective, not a trivial exercise but quite revealing. Reviewer #1 is correct that the terms dissemination rate (DR) and transmission rate (TR) both using these words and abbreviations have different meanings across articles. We have now place additional and explicit emphasis on this observation, with line numbers cited in the general responses. We also made a choice to describe these terms using the concept of stepwise and cumulative rates clearly defined in Yifei Wu et al. 2022. The only limitation of the proposed terminology is they did not propose short abbreviations, which we have done in Tables 1 and 5; we use IR (infection rate), stepwise dissemination rate (SDR), cumulative dissemination rate (CDR), stepwise transmission rate (STR), and cumulative transmission rate (CTR). We show which parameters were presented in each article (Table 1) and calculated, where possible, each of these rates in Table 5, even if the original authors did not. We believe that Table 5 currently, addresses Reviewer #1’s major concern directly and appreciate going through this exercise. Please see our general response above for additional details.

10. Line 433 (Table 4): How can 19 studies in the “Infection” column be only “20%?”

Response: Thank you for catching this error, it has been changed to 42%

11. Line 478 (Table 5): See comment 9 above. It can be extremely misleading if the data presented here are using different definitions of transmission. These rates need to be adjusted to a single definition. Note, using the percentage of mosquitoes with a disseminated infection is itself very misleading as mosquitoes with a very low dissemination rate may appear to have a very high transmission rate. Look at the table, how can a mosquito have a 43% infection rate and a 90% transmission rate?

Response: Again, see general responses about this issues and in particular revised Table 5.

12. Lines 508-509: Again, how can you have “significant transmission of ZIKV” with only a 42% infection rate?

Response: Removed significant.

13: Lines 529, 531: First of all, it is Ochlerotatus, not Ochelerotatus. More importantly, is it Ochlerotatus detritus or Ae. detritus? The genus Ochlerotatus was briefly establish for some of the Aedes species a number of years ago, but nearly everyone has gone back to using Aedes. Note, if you use Ochlerotatus, then it should also be Oc. taeniorhynchus, which I do not recommend.

*Response:* We have changed *Ochlerotatus* (misspelled) to *Aedes detritus*, throughout the manuscript, providing in parentheses that this species was originally reported as *Ochlerotatus detritus* in the original article or reference 70. We appreciate the suggestion.

14. Line 535: This is the second time that the authors mention that one of the reasons that Ar. subalbatus was tested was because they develop in human waste lagoons where ZIKV could be shed in urine. I believe that the study found no risk of the larvae becoming infected despite exposure to very high titers of ZIKV in a water/urine suspension in the laboratory. Repeating the statement above, without clarification that they didn’t find that risk, is misleading.

*Response:* We have added the following sentence to this section for clarification (Line 595-597).

“Furthermore, *Ar. subalbatus* larvae reared in water containing ZIKV and human urine did not result in any adult mosquitoes with detectable ZIKV RNA in saliva, providing no evidence of transmission via this route.”

15. Lines 575-579: Will warmer temperatures really affect ZIKV transmission? Remember, yellow fever and dengue were once common diseases in the United States, including Philadelphia, New York, and Boston. It was not warmer temperatures, but social economic status that allowed for the transmission of viruses by Ae. aegypti. However, warmer temperatures will allow for the greater and more efficient transmission of zoonotic viruses.

*Response:* We made the following modification to emphasize your point above, with new text shown in italics below.

(Lines 655-657). “As climate change increases average temperatures in temperate regions of the world, these species could grow in importance as secondary vectors of ZIKV (81), if the behavioral characteristics of the vectors and human costs favor contact between them.”

16. Lines 642-644: Yes, the mere isolation of a virus from a field-collected species does not mean that it is involved in the transmission of that virus. Even if a species is completely incompetent for a particular virus, if it had recently fed on a viremic host, it would contain virus. Similarly, when mosquitoes are sorted for virus detection, legs of numerous specimens break off and may adhere to the body of a mosquito of a different species. If that leg came from an infected mosquito, then whatever species it adhered to would appear to be positive. You might want to add a little about why the mere detection of virus from a species does not make it a vector.

*Response:* We made the following modification to emphasize your point above. New text in italics.

Lines 741-746: “Virus isolation from field-collected mosquitoes does not constitute evidence for their role as a vector, only that the mosquito fed on an infected host, or contamination occurring within a trap. Laboratory VC studies are required for vector implication to justify further studies on the role of these species in potential spill-over events as well as species whose ecology has changed and could have important implications for ZIKV transmission.”

17. Lines 703-708: Despite what was said in this paragraph, the use of the number of disseminated mosquitoes as the denominator is a relatively new use, and as I explained in comment 9, may be a very misleading number. If only 1/100 mosquitoes has a disseminated infection and that one transmits virus, then if only the disseminated mosquitoes are counted, the transmission rate for that species is 100%, making it appear that the species is very important, while if in another species, in which 100% develop a disseminated infection and only 20% transmit, that species would have a 20% transmission rate despite have 20-fold higher vector competence. This new definition came out of the Institute Pasteur, and I believe that they introduced it to make their work look more important, i.e., in the example above, a 100% transmission rate is much more publishable than a 1% one. From a public health standpoint, we need to know how efficiently a particular mosquito species is as a vector, saying that the species above has a 100% transmission rate might lead to the control of the wrong species. You sort of indicate this on line 717-718.

*Response:* Again, please see our general responses and response to comment #9. You are correct, although ACM thought the use of the terms efficiency to contrast stepwise and cumulative rates was well established, a second look at each of the articles argue to the contrary. In reading the literature, we share your concern that the definitions used are often manipulated (maybe too strong a term) to make for a “better story”. In the submitted manuscript we did try to provide some examples of this, but some of these comments were challenged by the reviewers. We now clearly make the point that cumulative rates are important for understanding public health importance (Lines 811-817). We use somewhat new terminology proposed by Yifei Wu et al. throughout the manuscript, as we believe it provides clarity and does not adopt any standard terms or abbreviations. In fact, it was somewhat frustrating that there has not been a general call within the VC community to propose standard terms that should be used moving forward, but clearly there is a reluctance to do so. For example, the ZikaAlliance has clearly adopted the terms efficiency, and honestly it is easy to understand and seems to be gaining some momentum. We take the opinion that both types of rates should be presented and considered moving forward.

From the general comments section above: We have added a paragraph describing the typical steps in a vector competence experiment to the introduction (see Lines 124-133). Then we describe the concept of stepwise and cumulative “rates” and define them (Lines 133-138). We address this issue more assertively throughout the manuscript and in results section added under vector competence parameters (Lines 446-449, **460-471**). The lines in bold directly address Reviewer #1’s deep concern about how there are multiple definitions for the terms dissemination rate (DR) and transmission rate (TR) used in the literature. We would like to avoid placing blame or any commentary on how this situation evolved or why, but strongly acknowledge that it is an important problem. Finally, we further emphasize this issue in the discussion (Lines 806-821, 823-834).

18. Line 733: What do you mean by “traditional methods?” the use of animal models and checking for infectious virus are the traditional methods.

*Response:* Changed traditional methods, to artificial blood feeding methods (Line 852).

19. Lines 734-736: Various studies have shown that the amount of virus injected when feeding on a vertebrate host is significantly higher as compared to the amount of virus injected during artificial methods to collect saliva. Similarly, transmission rates are usually higher to a vertebrate than when collecting saliva and testing it. Many laboratories don’t want to recognize this as it is difficult, or even impossible, for them to maintain the necessary animal colonies. Therefore, they use the less scientifically efficient artificial techniques.

*Response: We have modified that paragraph to the following (Lines 851-859).*

“The observation that using a viremic animal to infect mosquitos represents a more realistic model than artificial blood feeding methods is not a new concept (80,85). Financial and logistical limitations, especially in low- and middle-income countries where ZIKV and other arboviruses are endemic, do not have the facilities to use more realistic study designs. Thus, more detailed head-to-head comparisons of results from studies using artificial blood feeding with those using animal models or direct human feeds need to be conducted. The same is true for testing transmission from mosquitoes to a new host, that is – head-to-head comparisons of virus detection in saliva to infection of neonatal mice, especially under circumstances when mice become infected in the absence of a clear presence of infectious virus in saliva.”

20. Lines 769: Unfortunately, using viral doses that are consistent with viremias observed in humans in an artificial blood meal will result in significantly lower infection rates in mosquitoes than if they ingested the same dose of virus from a viremic animal (i.e., human). Therefore, studies using those doses would greatly underestimate the importance of that mosquito species.

*Response:* The sentence as written recognizes your point above. We are paraphrasing Azar and Weaver reference, who clearly state that achieving doses consistent with those observed in humans is easier to achieve with an animal model.

21. Lines 794-796: Yes, if the study only tested 15 mosquitoes and did not find transmission, that really doesn’t mean that the mosquito tested was incompetent. However, if they tested 10 specimens and eight of them transmitted virus by bite, then I think that they have clearly shown that this species may be very important.

*Response:* We feel that the following sentence “This would be particularly important for reporting results for species deemed negative” captures your sentiment above. We agree that 8 of 10 mosquitos tested would be convincing, and potentially appropriate for species that are difficult to move to the laboratory or difficult to capture in the field. We, like many authors before us (including Reviewer #3), are reluctant to define these standards in stone, but we believe that our grading tool needed to address this issue. It is not our aim to develop guidelines but suggest that subject area experts try to do so and all the issues you have raised in this review would be appropriate and important discussion points.

22. Line 812: I would be very surprised is there is any difference in infection rates determined by testing bodies or midguts, but it is a lot more labor intensive to test midguts.

*Response:* We agree that dissecting midguts is far more labor intensive that testing bodies and is not clear why several investigators choose to do so. We don’t know the answer to that question. If the laboratory methodology is sound and contamination is avoided, your assumption that there would not be a difference is likely correct. However, we are suggesting that clear comparative studies would be useful.

23. Lines 812-813: Heads and salivary glands are testing two different things. It is possible to have a disseminated infection, i.e., positive head and the salivary glands be negative. Likewise, a positive salivary gland does not mean that there will be virus in the saliva.

*Response*: Back to point #22. We ask why dissected parts versus whole parts. See modified text (Lines 927-941).

There is a strong need for future research that establishes what are significant differences between experimental approaches such as the use of dissected body parts (midguts, salivary glands) compared to whole body parts (heads, legs+wings, bodies) to evaluate infection and dissemination, and the impact of using murine models as well as other artificial feeding systems on VC parameters, and importantly, to develop large collaborative multi-country projects possibly taking advantage of research networks (eg. ZikaAlliance) (79), Centers for Research on Emerging Infectious Diseases (86) to conduct large scale simultaneous evaluations of specific mosquito species with common protocols to appropriately address the inherent geographic variation in both mosquito and virus strains..

24. References: These need to be formatted properly.

a. Only the first word and proper nouns in a reference title should be capitalized. See references 13, 36, 39, and others.

b. References should not be in all caps. See reference 20.

c. Shouldn’t the journal for reference 28 be “Trans R Soc Trop Med Hyg” instead of “Transactions of The Royal Society of Tropical Medicine and Hygiene?”

Response: Thank you. We have reviewed all the references.

Minor Comments:

25. Lines 49-50: Should “…identify knowledge key gaps” be “…identify key knowledge gaps?” Done

26. Line 59: Why is there a “comma” after “quinquefasciatus?” Done, and removed

27. Line 98: “Aedes” needs to be written out the first time it is used in the body of the manuscript. Done.

28. Line 103: Traditionally, numbers less than 10 are written out, so this should be “eight genera…” Note, it appears that there is an extra space after “genera.” In contrast, all numbers used in measurement are given as numerals. Therefore, on line 149, “five years…” should be “5 years…” Please check the rest of the manuscript and correct these. Done and thanks for the grammar tip.

29. Line 165: Why is there a space after the “)” and before the “comma?” done

30. Line 169: Should “WHOLIS” be “(WHOLIS)?” done

31. Line 191: Should “wild mosquitoes” be “field-collected mosquitoes?” done

32. Line 203: “n= 8.986)…” should be “(n= 8,986)…” Note that a comma was correctly used elsewhere. done

33. Line 291: “Medical Entomology [3] and” should be “Medical Entomology [3]), and” with both a close parenthesis and a comma. done

34. Line 330 and many others: Why write out “Zika virus” when “ZIKV” was established and used many times earlier in the manuscript? done

35. Line 371: Why are Aedes, Culex, Anopheles, etc. written out each time they are used in this table? They should only be written out the first time that they are used. Although we had some disagreement on this issue among the team, we follow your suggestion.

36. Line 377: Why is the “10” in “log(10)” in parentheses? Changed to log_10_ PFU/ml

37. Line 380: “…ten also…” should be “…10 also…” done

38. Line 393: “7 studies” should be “seven studies.” See also lines 452, 475, and others. done

39. Lines 440-441: Why is “(Coffey et al. 2018)” listed here? Should this be assigned a reference number? Note, all of the reference numbers may need to be adjusted. done

40. Line 441: Why is “Sabethes” written out throughout the manuscript? You don’t write out Aedes or Culex? These should all be “Sa. species” as Sabethes was established on line 376. The same is true for Armigeres. done

41. Line 443: Again, reporting a titer to two decimal places is reporting data to a level that is not accurate. Yes, I know that some papers do this, but that does not make it right, and you should not make the same error. On this line, simply report the titer as “6.8 log10 PFU/ml.: Note, elsewhere in the paper, PFU is in all caps. Please be consistent.

Done (see general responses). We have attempted to standardize the reporting of titers and only use one decimal point. Hope we caught them all.

42. Line 484: Change “vector were…” to “vectors were…” done

43. Line 501: What does “more consistent infection rates” mean? Dose that mean that the rates were consistent, i.e., 5%, 6%, 4% in three separate trials or that they were consistently higher than those for Ae. aegypti tested from that area?

Response: (Lines 567-570): See modified text below.

In contrast, a later study showed two populations of *Ae. polynesiensis* from French Polynesia and Wallis and Futuna high infection rates that were less variable than *Ae. aegypti* populations from the same region and showed high dissemination rates, and low transmission rates (< 10% at 21 dpi) (57).

44. Line 515: Why is there a “comma” after “injection,?” comma eliminated.

45. Line 516: “Colorado USA…” should be “Colorado, USA…” done

46. Line 520: “Ae. triaseriatus” should be “Ae. triseriatus” done

47. Line 521: Why is “(Hart et al. 2017)” listed as a reference by name rather than by reference number 51? done

48. Line 531: Change “or Ae.” to “nor Ae.” done

49. Line 624: Why “(2018)” rather than “(57)?”done

50. Line 766: Why “(2019)” rather than “(79)?”done

Reviewer #2: In the review “Secondary vectors of Zika Virus, a systematic review of laboratory vector competence studies” Bisia and colleagues have conducted a thorough and transparent review of the existing Zika virus vector competence literature with respect to laboratory studies conducted on potential secondary vectors. As a laboratory construct, vector competence represents the research community’s best method to examine a complex interplay of factors that contribute to a given mosquito species or populations ability to serve as vector for a given agent. That said, the methodology/discipline suffers from a pervasive lack of unity with respect to methodologies, definitions and other critical parameters. In conducting these analyses, Bisia et al are able to illustrate this issue alongside their primary objective of identifying potential secondary vectors for ZIKV. Given the increased incidence in the emergence of zoonotic diseases, including arboviruses, increasing the rigor and reproducibility in this discipline is paramount. This manuscript illustrates this in the context of the ZIKV outbreak in the Americas from 2015-2018.

In total the manuscript is well-written, articulates its methodologies clearly and transparently, identifies promising avenues for further development, and draws appropriate and scientifically sound conclusions. Publication of this manuscript is therefore highly recommended albeit with the following suggestions and comments put forward for consideration.

*Response: Thank you.*

Major Points

1. Lines 141-142 and throughout: Something to note; the connection between Culex quinquefasciatus and ZIKV was NOT initially facilitated by peer-reviewed research. A group that was affiliated with public health agency FioCruz in Recife issued a press release (e.g. <https://www.theguardian.com/world/2016/mar/03/zika-virus-carried-more-common-mosquito-scientists-say>) in the absence of public data a full year prior to the publication of their manuscript.

*Response:* Excellent point, and we added a reference to this (Lines 750-753). ACM also saw the group’s presentations during that period and the final publication was quite flawed. But for us the publications by Guo et al., remains that one piece of evidence that can not be easily discounted, although MacLeod and Dimopoulous do a reasonably good job. Good example of a study that did not use an infectious assay, but transmission to neonatal mice is very difficult to argue with, unless you accuse the authors of fraud, saliva results were not consistent with transmission.

2. In the text and in the grading tool (Supplemental Table 1), the authors refer to the use of “low passage virus” as a parameter. While considering colonization of mosquito populations, authors define the association between the point value and the number of generations in the laboratory. I feel that a defined passage number should be indicated in the table, as “low passage” in the absence of a defined number is ambiguous.

*Response:* Agreed and we have now explicitly stated ≤ 20 passages (see page 27-28, Line 416 and Line 428); supplemental table). Additionally, in the section “Virus strains used”, we have added the following text to address this concern.

Lines 426-431. “Of the articles providing passage numbers, for most specific values that were generally < 10 passages for “low” passage, apart from one research group in Senegal reporting the use of 20 passages for strain MR766 (38). This latter prototype strain was reported to have between 146-150 passages for most authors who used it in their experiments (37,43,46,53,78). No recombinant viruses were used for experiments in the studies reviewed.”

a. Related; do the authors distinguish between passaged primary isolates or recombinant viruses in these experiments? Can this be noted in the text either way?

*Response:* No recombinant viruses were used in any of the reviewed article. We now state this implicitly on Lines 430-431 (see text in last response, last sentence).

3. Lines 760-761: The authors note the lack of experimental replication in the vector competence literature. Although the authors note that this is understandable due to the labor cost of these experiments, it also worth noting that from a strict standpoint such replication is not technically possible. Exposure of a given laboratory population is associated with a given generation of colonization (e.g. F3, F4, etc). In the case of mosquitoes that do not lay eggs that can undergo desiccation and storage such as Culex spp, it is logistically impossible to perform an exact replica of an experiment with the same generation of mosquito from the same population. While such experimental replicates ARE technically feasible in the case of some mosquito species, it also requires substantial up-front effort to rear large quantities of mosquitoes and store substantial cohorts of eggs to allow for generation matching.

*Response:* There was agreement on this issue amongst all three reviewers, in particular reviewer #3. We agree with your arguments above but note that in a few studies biological replicates were conducted and we think there are some important messages from those examples: 1) there is a lot of variability among replicates, even if they are carried out simultaneously, and maybe even more if not. I think it is important that readers see how messy VC data can be. It is not pretty, and any interpretation needs to be made through that lens; 2) The issue of statistics (not just the calculation of rates) is frustrating, especially when some of the rate data presented is based on very low sample sizes. See the following modification now on Lines 882 to 892.

“Also apparent was the lack of experimental replication, which can only be accomplished if sufficient insectary space, mosquitoes, and labor are available to run replicates at the same time, all of which presents numerous technical challenges of rearing enough mosquitoes of the same generation to conduct these experiments. Laboratory conditions cannot be 100% duplicated, running multiple replicates for distinct species/virus or mosquito strain may be less of a priority that other components on the quality grading list. Related to this issue, however, is the observation that few manuscripts provided measures of variability or conducted statistical analyses, a more serious issue. Because our review focused on secondary vectors, the use of multiple virus and mosquito strains was limited, but either additional geographically isolates or studies that include a wide range of geographic mosquito strains together with multiple virus strains will provide convincing evidence of a species VC (79)”.

Minor Points

1. Line 138: This may be pedantic; however, I believe this sentence requires clarification. Specifically, the phrase “much of our knowledge comes from…” seems inappropriate as our current knowledge base far surpasses what was known solely based on the YFV surveillance. Corrected the sentence.

2. Line 483: The word “both” on this line appears to be misplaced as there is no secondary factor after “laboratory evidence”. Deleted the word “both” in this sentence.

3. Line 491: The correct spelling is Sabethes cyaneus 4. Lines 495 and 497: The authors here refer to Aedes polynensis, which is a misspelling of the species name Aedes polynesiensis. done

5. In line 576, the authors identify Ae. japonicus, Ae. vexans, “and to some degree” Ae. detritus as potential secondary vectors given higher temperatures. In lines 659-661 the authors note that Ae. vexans, Ae. japonicus, and Ae. polynesiensis as possible candidates for follow up studies. Please correct for consistency. We added the *Aedes detritus* mosquito as a vector for future studies and deleted *Ae.* *polynesiensis*.

Reviewer #3: See the attached review report (note we pasted the text in here.

In the manuscript entitled “Secondary vectors of Zika Virus, a systematic review of laboratory vector competence studies”, Bisia *et al*. present the result of a systematic literature review of *Aedes* vector competence for Zika Virus (ZIKV), conducted on a myriad of scientific articles, extracted from the most relevant search engines (PubMed, Cochrane Library, LILACS, Web of Science, WHOLIS, Google Scholar).

Vector competence (VC), an evaluation of the vector capability to transmit a pathogen, provides a summary statistic for a two-fold process: i) the ability for a vector to infect itself upon feeding on contaminated blood, and ii) its subsequent ability to infect back another host (human, in the case of ZIKV). To be fully potent for that second step, viral particles in the mosquito are required to breach multiple barriers, to travel the midgut all the way to salivary glands.

The key findings of the paper are:

1) The large heterogeneity in reported outcomes and terminology, along with the difficulties this causes to integrate results into broader studies such as meta-analyses,
2) Identification of *Ae. japonicus* and *Ae. vexans* as potential vectors of interest ZIKV spread,
3) Overall poor VC for *Culex* *quinquefasciatus*.

The authors also identify critical methodological issues that may hamper the generalization of previously-published results.

**Overall, the article provides a good summary of the current state of scientific research for ZIKV vector competence, and I believe it would be of benefit to the readership of PLOS NTDs provided the author address the comments summarized below**. In particular, the introduction is carefully written and does an excellent job at detricating *vector competence* from *vectorial capacity*, two notions that are often mistakenly used. The methodology used for the systematic review is sound and the research question well phrased in the first sentence of the Methods section.

My main concern for this article revolves around the timing for publication: the literature search was stopped as of March 15^th^, 2022 (more than a year ago at the time of writing this review) and since then, at least one major publication that corresponds entirely to the “future work” authors are calling for has been published (Obadia *et al.*, <https://doi.org/10.1038/s41467-022-32234-y>).

*Response:* With any systematic review a search stop date must be selected and ours was March 15, 2022. It took a long time to extract all the data from each article and develop the quality grading tool, which both MS and ACM, the two researchers who did the data extraction and grading would agree should be improved upon by subject area experts. The grading tool would better serve as a checklist guidance document. Resisting the temptation to whine, effectively extracting all the information we suggested in the grading tool was frustrating to say the least. Thanks to a push from all three reviewer’s all the information in Tables 1, 3 and 5 has been double checked. It took time and to include the article you suggested we would need to essentially start the study over again. However, your article is now highlighted in the discussion of exactly how future studies should be conducted as well as for other purposes.

# Major comments

**Major comment #1**

My first obvious comment arises from the “Interpretation” section of the abstract: I fully concur that future research initiatives are needed to develop vector competence laboratory experiments standardized across countries, in an effort to properly characterize VC while accounting for Gene x Environment factors. In fact, in Table 1, several of the selected publications were from teams associated with the ZIKAlliance consortium (<https://zikalliance.tghn.org/>), a Horizon 2020 European research program. The same consortium published in August 2022 a consolidated analysis of such a standardized effort, by studying 12 countries, 50 mosquito populations from 6 different species, including sampling at multiple locations in many countries. *Aedes* and *Culex* mosquitoes were among those studied, while 6 ZIKV genotypes were used for challenges. The findings are in general agreement with the present literature review regarding the concern for *Ae. japonicus* and the lack of susceptibility for *Culex*. I don’t understand why the authors stopped their literature review as of March 2022, but this study definitely addresses some of the “future research initiatives” that are called for.

*Response:* Please see our previous response on the reason the search was stopped in March 2022. We have changed the Interpretation section in the abstract to the following (Line 66-68) as we highlight the ZikaAlliance initiative in our discussion.

**“Conclusion:** Recent, large collaborative multi-country projects to conduct large scale evaluations of specific mosquito species represent the most appropriate approach to establish VC of mosquito species.”

Why did the author stop including articles as of March 2022 ? We are now a year later and literature has apparently evolved on that topic, which would make some statements (see just above) partially addressed.

Response: Please see general responses section.

This comment overall applies in the discussion as well (L625–627).

Response: We can state that the search for new articles was stopped to initiate the retrieval, examination, and analysis of the discovered papers. The duration of the process was lengthy due to the vast quantity of papers found, specifically the 45 papers that necessitated information extraction and also the quality assessment tool that demanded meticulous attention to detail. We do refer to Obadia et al. 2022 in the following parts of the manuscript.

Discussion: Lines 756-765

“Obadia et al. (79), a study published a few months after our end search date, provides an illustration of a coordinated research effort using a unified protocol across a wide geographic distribution using strains of *Ae. albopictus* and *Ae. japonicus* directly from the field involving multiple research groups. These kinds of efforts allow experimental studies that would be otherwise logistically impossible for a single research group. A collaborative approach where multiple research groups evaluate 1 or 2 local mosquito strains and if possible, an epidemiologically relevant virus strain (e.g., in sites with endemic transmission a strain from the same location), provide more credible and robust conclusions.”

Discussion: Lines 818-821.

“One alternative has been the introduction of the terms dissemination efficiency (DE) and transmission efficiency (TE), terminology used in eleven of the reviewed articles but clearly distinguishes between stepwise and cumulative rates (79).”

Discussion: Lines 887-892.

“Related to this issue, however, is the observation that few manuscripts provided measures of variability or conducted statistical analyses, a more serious issue. Because our review focused on secondary vectors, the use of multiple virus and mosquito strains was limited, but either additional geographically isolates or studies that include a wide range of geographic mosquito strains together with multiple virus strains will provide convincing evidence of a species VC (79).”

Discussion: Lines 937-9341.

“to develop large collaborative multi-country projects possibly taking advantage of research networks (eg. ZikaAlliance) (79), Centers for Research on Emerging Infectious Diseases (86) to conduct large scale simultaneous evaluations of specific mosquito species with common protocols to appropriately address the inherent geographic variation in both mosquito and virus strains.”

**Major comment #2**

The scoring system used for the “Quality assessment” is at the core of grading the 45 studies that made it into the literature review. I can only appreciate how complex building such a scoring system is, and that some decisions have to be made eventually. The discussion however lacks a section debating such decisions and the relevance of the scoring values. I note that the two scores (total score (TS) and methodology score (MS)) correspond to the total sum of items and that no weight is applied. This is partly compensated by the DELPHI-like approach where two researchers from the team scored independently each study and debated until reaching consensus. This however does not challenge the weights given to every item. Furthermore “reaching consensus” may be viewed from a mathematical aspect as a sort of average, though between-investigator variation is an important source of noise that should be considered. At minimum, some more explanations about the procedure in case of discrepancies is warranted:

- Are there specific articles that made it challenging to reach consensus consensus? If so, which ones, and how as this handled?

*Response:* Reaching consensus was not a problem overall. For most of the responses, especially the components of the MS, the scoring rules were well defined and not subjective, but finding the needed information within the manuscript could be quite a fishing expedition. Also, because the group were not subject area experts, per se (ACM has some experience), familiarizing we had a learning curve with the laboratory terminology. Consensus was often as easy as one reviewer pointing out in the manuscript, the infectious assays used, generation of strains of mosquitoes or virus used. There were items that we would exclude moving forward, for example vector competence in the title, hardly seems relevant. The issue of study limitations also generated significant discussion. MB felt all articles should include a discussion of limitations, whereas, ACM emphasized transparency about design limitations, especially for studies that did not include infectious assays. In this case ACM adopted a more literal and consistent approach. The major concern that both ACM and MB felt that it was an onerous and time-consuming task, that did not seem practical in the end. Under PRISMA guidelines, reviewed articles should undergo a quality assessment, and we attempted to device a tool that would 1) identify articles that should not have been published and thus not included (no article met this low bar), and 2) provide grades of evidence, analogous to those reported in Wilson et al. 2015.

Are there specific items that showed consistent variations across grading researchers? This would imply differences in interpretation and would make the scoring system difficult to rely on for that item.

Response: No, except for what we described above. We do not want readers to focus on scores, and debated if we should include scores in Table 1. It is important to understand what highlight in your next comment, some study designs are stronger than others. Our tool may function better as a checklist for authors. Note the figure to the left is now cited on line 679. (Reference 83)

L673–674 in the discussion also calls for that comment. There is no point in “rewarding” another article but rather develop a scoring system that provides quantitative insight about a paper’s methodology.

Response: Removed the word rewarding.

Changes to the manuscript to address Major comment #2. Overall, we provided more discussion and description of the Quality Assessment Tool. Key text additions are shown below.

Methods Section (Lines 285-295):

“If the scores did not match, all 38 scores were compared and where differences were observed discussed between the two researchers. For most scores that were not concordant, the article was reviewed to confirm that each researcher had correctly extracted the information necessary to provide a score (e.g., type of laboratory assay, mosquito colony history, virus passage history, number of virus or mosquito strains used). Scores were adjusted based on careful review of the manuscript and the score justification provided by each researcher. For some scores that were more subjective (e.g., clearly written abstract, adequate discussion of study limitation) the researchers discussed their scores, compared them to other articles to standardize their approach to each question. Each score was discussed until both researchers agreed on a single score. No studies were excluded after the quality assessment, but the analysis and the report of the results considers this quality assessment.”

Results Section. Quality grading tool assessment. (Lines 331-341): We have removed Figure 2 at the request of Reviewer #1, indicate standard deviation (± SD), and added the following descriptive sentence.

Lines 339-341 “Interestingly, of the seven publications in short format (letters, dispatches, short communications), five (71%) scored in the lowest 25%, in large part because important metadata was excluded.”

Discussion Section. We have added the following paragraph (Lines 672-688)

“Although, we developed and applied a quality assessment tool as part of our systematic review, its application proved cumbersome. First, not all the reviewed manuscripts provided data sets either posted online or in a data repository. New studies will be required to do so, and this requirement will help overcome problems associated with inconsistent reporting of study metadata. The purpose of our QAS tool was not to rank articles, but to clearly delineate minimum standards, and distinguish between different levels of evidence quality. As an analogy, for field trials, data from randomized controlled trials is seen as the gold standard, followed by non-randomized controlled trials, and finally observational studies (83). For VC studies, those using infectious laboratory assays, with higher sample sizes (including replication and statistical analysis), a wider range of mosquito and virus strains, and clear measure of transmission represent higher quality data than those that only detect RNA, have smaller samples, and are limited by less natural conditions using colonized mosquito strains and heavily passaged viral strains, but these later studies can be informative is reporting is transparent and study limitations are understood. Our grading tool could not distinguish between well designed and executed studies where the reporting was limited (e.g. short format articles) and poorly designed studies. We encourage subject area experts to modify this tool if it is to be applied for future systematic reviews”.

Reference 83= (Wilson et al. 2015: <http://dx.doi.org/10.1016/j.pt.2015.04.015>), same as table above.

**Major comment #3**

As a follow-up comment about that scoring system, Figure 2 provides the distribution of TS and MS indicators. This figure does not read well and would benefit from better plotting in a true histogram-like manner (where the area of each rectangle would reflect on its probability). The boxplots are again hard to read (median and whiskers being hardly distinct from the background color). Putting MS and TS side by side lead the reader into thinking these two distributions are meant to be compared, though they aren’t. My suggestion would be to rethink that figure to show the overall distribution using only one panel (either combining histograms and a horizontal boxplots, or simply a histogram), possibly faceting by type of score. The aesthetics should be enhanced, too (font size, axis labelling etc.) In the corresponding section of the text (L301–309), please also indicate what the error margin relates to (“the mean score was 29.0 ± 4.2”: what is that 4.2 value?). Looking at the distributions in Figure 2A, there are evidence of bimodal distributions around lower and high end of the possible score range (e.g. 2 gaussian distributions in each score). This is in line with my previous concern for the need of debating the relevance of the retained scoring mechanism: I would rather expect one single gaussian for each score, as in its current state this points towards difficulties for reaching an “average-level study”.

Response: Please see our response to Major comment #2. We have removed Figure 2 and thing the bimodal distribution you observed reflects that most of the reviewed articles were of high quality, with a small group with lower scores were represented by manuscripts that only used PCR to detect ZIKV and short format articles that had scant methodological details, the latter demonstrating a weakness of the QAS tool. Additionally, neither of the researchers who did the scoring (MB, ACM) loved the process and it was more effort than appropriate. We did indicate that we were presenting standard deviation (SD). This tool should be viewed more as a checklist, rather than a tool to rank articles. The QAS should not become the focus of the article, rather we describe clearly possible secondary vectors which clearly not that significant epidemiologically and highlight the issue of striving for more standardized and consistent reporting of this kind of data.

**Major comment #4**

In the paragraph “Review of evidence for individual vector species other than *Ae. aegypti* and *Ae. albopictus*”, the authors identify 4 context of epidemiological relevance. I believe some of these (especially #1 and #2) would correlate with the usual definition of occupational (#1) vs. peri-domestic (#2) exposure to mosquito bites. Mentioning the context for mosquito exposure would be useful as an epidemiological descriptor for the population at-risk. Furthermore, there are 4 categories identified here though 5 are detailed in Table 5: presumably, the “Other” section was added to include the *Anopheles* species? Please modify the main text to help identify which category corresponds to which in Table 5.

Response: Table 5 has been modified significantly based on suggestions from both Reviewer #1 and Reviewer #3. Table 5 now includes 4 sections: Forest, Local, Widespread Temperate, and Cosmopolitan. We respectfully disagree that the forest species category solely represents occupational exposure, but rather a zoonotic virus maintenance cycle. This category will be of interest to those interested in spillover of virus from an animal cycle to human and could become interesting as human communities penetrate these forest cycles. Yellow Fever outbreaks associated with *Ae. africanus* and other canopy vectors have been documented in West Africa. Interest in these species is that they can also adapt to more *Aedes aegypti* like behavior, generating new kinds of transmission cycles in the future (see Lines 748-753 in the discussion). We now include *Anopheles* in the “local” category but highlight species in the Widespread temperate and cosmopolitan categories, due to the wider distribution of these species. We provide important details now for *Ae. detritus, Ae. japonicus, Ae. vexans* where multiple studies were carried out highlighting the important role of temperature (future comment). We have reorganized the text so that if flows with the species listed in Table 5.

Lines 743-748

“Laboratory VC studies are required for vector implication to justify further studies on the role of these species in potential spill-over events as well as species whose ecology has changed and could have important implications for ZIKV transmission. For example, *Ae. africanus* and *Ae. furcifur* became important vectors of yellow fever in villages where these species moved freely between the forest canopy and villages nearby where *Ae. aegypti* was absent. Theoretically, the same is possible for ZIKV.”

**Major comment #5**

In the discussion (L591–592), the authors emphasize the need for reporting standards. A natural follow-up to that systematic review would be a meta-analysis of infection/dissemination/transmission outcomes. The authors should consider mentioning how study heterogeneity can be estimated should such an approach be taken (Cochrane’s Q, Higgins’ and Thompson’s I^2^, among others).

Response: You are correct that a natural follow up to a systematic review would be a meta-analysis, and perhaps for *Ae. japonicus, Ae. vexans, and Ae. detritus.* But the number of studies is limited. Now for *Ae. aegypti* and *Ae. albopictus* a meta-analysis would be quite justified. We looked up the heterogeneity tests you suggest and if we had the statistical skills, we would have taken the next step to conduct a meta-analysis (but in many ways, these Nature communications papers is that type of analysis). The number of papers of the previously mentioned species (not *Ae. aegypti* or *Ae. albopictus)* are still low and probably do not sure justify a meta-analysis. To conclude, a metanalysis is probably not well justified and well beyond the scope of our article.

# Minors comments

**Minor comment #1**

The section “Time and geographical clustering of studies” is useful to summarize study contents but would be well supplemented by a visual aid so that the reader could quickly realize how well the selected studies cover the areas of interest for ZIKV. Could the author come up with a global map detailing at the continent(country)-level how many studies apply to a region and how these studies are split across the three-time windows (pre/per/post ZIKV epidemic)?

Do you agree to provide a map?

Response: We like your idea and have developed a figure the new Figure 2 on page 24 of the manuscript (Line 356-358)

**Minor comment #2**

The ”Vector competence parameters” section does not discuss the temperature used across experiments. Temperature (and, somewhat related, altitude) has been shown to positively correlate with infection/dissemination/transmission efficiency through various biotic and abiotic processes (see Bellone *et al.*, <https://doi.org/10.3389/fmicb.2020.584846>; Mercier *et al.*, <https://doi.org/10.1038/s41598-022-10977-4> though the latter does not focuses on ZIKV but other arboviruses). Surely, among the 45 studies, this important parameter was often reported and integrating it would be useful in that section. Likewise, the time since inoculation is hardly mentioned although it is often positively associated with the outcomes of interest.

Response: Good observation and here is what we did.

Table 1 (now includes the rearing and when available laboratory condition for each experiment).

Table 5 (experiments that varied temperature are reflected in the table).

Text: Added a final paragraph to the results section (Lines 641-647), and we comment on its positive association with infection, dissemination, and transmission.

Lines 643-649

**“Role of Temperature.**

Four of the reviewed articles conducted VC experiments that asked specific questions about the impact of rearing temperatures on European *Culex* species (52), *Ae. detritus* (70) and *Ae. japonicus* (59,73). Transmission increased with rearing temperature for both *Aedes* species tested and when *Ae. japonicus* was reared at more realistic fluctuating temperature scheme (daily variation between 14°C and 27°C compared 27°C) there was no impact of fluctuating temperatures on VC (Table 5).”

**Minor comment #3**

The last paragraph of “Studies using mice” is sometimes difficult to read (esp. L452–461).

Response: we rewrote this section (lines 486-493)

“In six studies, mice were utilized (either neonatal or genetically modified) to infect mosquitoes in a more natural and efficient manner or to confirm transmission of virus from the mosquito to a new host. Vector competence parameters were higher for mosquitoes fed on mice than those using artificial feeding strategies or direct testing of saliva, consistent with the observation that blood from viremic animals are typically more infectious for mosquitoes than artificial meals (80). Four studies used interferon deficient mice infected with ZIKV to infect *Cx. pipiens* and *Ae. triseriatus* (39), *Cx. quinquefasciatus* (51), *Cx. tarsalis* (61) and *Sa. cyaneus* (60) mosquitoes experimentally, compared to *Ae. aegypti*. All species infected using mice were unable to transmit ZIKV, except for *Sa. spp.*, where a single mosquito (11%) that fed on a mouse with 6.83 log_10_ PFU/ml after being held for 21 days tested positive for ZIKV by plaque assay, compared to 70% of *Ae. aegypti*.”

**Minor comment #4**

Table 5 is a very useful summary of all the paragraphs looking at evidence for vector competence by species. It however lacks description of how transmission efficiency (TE) is calculated. Judging by the low proportions, I’m inclined to believe this corresponds to the number of mosquitoes with viral particles in saliva divided by the total numbers of mosquitoes challenged. The TE column is also not explicit : does it correspond to the range of values in the Transmission column ? Please add detail in the legend. The “NT” abbreviation is mentioned in the legend but not used in the table (variations: ND, NT, Not tested). That table needs to be formatted slightly better.

Response: Please see the updated Table 5 and table legend and responses to Review #1 comments #7 and #19. Yes, the table required significant modification.

**Minor comment #5**

L530 : This is not unexpected as increasing TE with temperature has also observed with other arboviruses, too.

Response: Indeed, this is true.

**Minor comment #6**

The two paragraphs for *Ae. japonicus* and *Ae. vexans* (L547–552) appear in a very unexpected manner. Consider integrating into a single paragraph identifying vectors of potential interest esp. in settings as #3 (L469) ?

Response: We have reorganized these paragraphs.

**Minor comment #7**

L558–559 : please clarify the sentence.

Response: We rewrote this section to address both the sentence and address minor point #8.

**Minor comment #8**

In the paragraph “Possible role of *Culex* species”, the overall consensus is that *Culex* is hardly competent to transmit ZIKV, except for the (unproven) possibility of geographically isolated species. I believe this paragraph would benefit from a stronger phrasing of that overall *Culex* poor vectorial competence (in particular L566: I do not believe that these studies overall indicate that “*Culex* species [are] able to transmit”, but rather they show a very poor vector competences overall).

Response: (Lines 624-642), show below.

“A total of 24 of the 45 studies identified in our systematic review tested *Culex* species; of these 22 included *Cx. quinquefasciatus*, and 14 used mosquito strains recently brought from the field to laboratory. Thus, the overwhelming weight of evidence is that *Culex* species are unable to transmit ZIKV. *Culex* *quinquefasciatus* species were refractory in 10 laboratory studies using mosquitoes from colonies (n=5) and field (n=5). Of the remaining 11 studies infection rates were very low, dissemination rare, and one report on infectious saliva in one mosquito infected at very high virus titer. Three studies make claims that *Cx. quinquefasciatus* can transmit ZIKV. Guedes et al. (31) showed viral particles in salivary glands by electron microscopy, suggesting this was evidence of transmission, but was not able to demonstrate infectious virus in saliva. A study from China (30) showed transmission of ZIKV from laboratory infected mosquitoes to mice, however saliva from the same mosquitoes, tested by PCR only, showed presence of RNA at 8 dpi (80%) and 12 dpi (10%) but not 16 dpi putting in question the presence of infectious virus in the saliva. Finally, infectious virus was detected on FTA cards exposed to laboratory-infected field mosquitoes, evidence of transmission (32). In summary, the studies evaluating *Culex.* laboratory competence indicated that *Culex* species has poor VC for ZIKV overall, but the possibility of geographically isolated strains that are competent must be considered. Macleod and Dimopoulos (13) provide a comprehensive review and interpretation of the data available for *Cx. quinquefasciatus* VC to date.”

**Minor comment #9**

In the discussion (L629–633), the authors highlight that science may be rushed in times of uncertainty. History has repeated itself at an even larger scale over the past few years. This is not unusual and, in my opinion, shows a strong mobilization. If the aim of that paragraph is to emphasize that early, rapid papers should be interpreted more cautiously than those published a few years later, this message should be made clearer.

Response: See modification made to this section of the manuscript (Lines 722-736). A major issue is the short publication format, and we try to describe the “trade-off” in the text below. Our frustration from these early papers, was more about the studies being less relevant, for example *Anopheles,* because it was available in the lab. There is nothing wrong with this, but it does provide the perception that some folks were just jumping on the band wagon for a quick publication. We recognize this might be a bit harsh, and now provide a more nuanced discussion. We don’t feel justified making a statement about that perhaps the Guo et al. manuscript should not have been published as is and should have been made to at least retrospectively (they kind of tried), show the presence of infectious virus in their saliva samples. As reviewer #2 points out, a large part of the problem at the time was some reporting of data before peer review, which helped fuel the speculation about *Culex quinquefasciatus* being involved. This was where we were attempting to go, albeit not effectively.

“At the initiation of the South American ZIKV outbreak, VC studies appeared to be designed based on what mosquito colonies and virus strains could be obtained rapidly. Many of these initial publications, were short communications, and in many ways appeared rushed. There was considerable concern in Europe and the US, that ZIKV could potentially be transmitted by local species, leading to a higher proportion of studies from these countries. Although accessing the risk of potential vectors in these locations made sense, early studies used mosquito strains that had been in colony often for decades, transitioning into more studies that collected mosquitoes directly from the field that could be brought back to the laboratory. Also, reading between the lines were studies that appeared to retrospectively verify they were detecting infectious virus rather than relying exclusively on PCR to detect ZIKV RNA. Thus, these initial publications often lacked detailed metadata that are important for future meta-analyses or full assessments of a particular species vector potential. These studies did provide needed quick assessments for making Public Health decisions. ZIKV represents a prototype of what occurs during the initial response to emerging Public Health Emergency, in which an unprecedented amount of research was published very quickly (82).”

**Minor comment #10**

L728: please detail the qualitative differences the authors are referring to.

Response: This paragraph has also been transformed based on other review comments now seen lines 851-859.

“The observation that using a viremic animal to infect mosquitos represents a more realistic model than artificial blood feeding methods is not a new concept (80,85). Financial and logistical limitations, especially in low- and middle-income countries where ZIKV and other arboviruses are endemic, do not have the facilities to use more realistic study designs. Thus, more detailed head-to-head comparisons of results from studies using artificial blood feeding with those using animal models or direct human feeds need to be conducted. The same is true for testing transmission from mosquitoes to a new host, that is – head-to-head comparisons of virus detection in saliva to infection of neonatal mice, especially under circumstances when mice become infected in the absence of a clear presence of infectious virus in saliva.”

**Minor comment #11**

L783–784: I do not understand the reluctance of authors for “small number of occurrences” (this happened earlier in the text but did not catch my attention until the phrasing I’m reporting here). First, the phrasing here is provocative and uncalled for. Second, rare events can be studied using appropriate statistical methods, the first coming to mind here being exact Binomial tests. In the case of *Culex*, how would one be able to characterize low/nil transmission rates other than reporting 0, 1, 2 mosquitoes out of more?

Response: we have eliminated the following sentence:

“Is it reasonable to include rates based on 1-2 mosquitoes from a single replicate in any future metanalysis.”

Similarly, L794, I respectfully disagree: 1 mosquito out of 69 is **clearly** way below the 20 infected *Aedes* out of 20 (100%) and also below the 14 (70%) that had disseminated the virus. The whole point of a title is to summarize the key findings in context. What is the point of that comment?

Response: We have modified this paragraph and eliminated the reference to the Karna paper now in lines 915-926. As you point out, 1/69 transmission rate is low compared to *Ae. aegypti* (not zero), but that single mosquito was positive in all compartments, so IR=DR=TR (any way to measure it), which is a bit weird in its own right. When the conclusion of the manuscript is based on a single mosquito, it is difficult to have full confidence in the result. If the experiment were repeated an similar results were observed. Using jargon, it feels like a one off, not wrong, but difficult to have complete confidence. We have removed the objectionable comment.

**Other minor suggestions**

L195: typo in the word “competence”. done

L262: typo in the word “appropriately”. done

L335: de-italicize “were tested”. done

L411: the verb is missing. Done

L418–419: a verb might be missing, or sentence should be rearranged? rearranged

L420–421: check syntax for last sentence of the paragraph. done

L610–614: please rephrase. done

L616–617: how are these data “misleading”? That sentence reads very critical and does not add a lot of value. I believe that no data is often better than poor/biased/unclear/… data. Maybe delete the last sentence?? okay

L633: typo in the word “assessing”. done

L656–658: please clarify what the word “strains” refers to, as it seems it’s used interchangeably for mosquito species, geographic location of mosquito sampling and ZIKV genotypes. done

L698: typo in the word “assessing”. done

L769–772: overlong and unintelligible sentence, please rephrase. done
