# Supplementary material for: Secondary vectors of Zika Virus, a systematic review of laboratory vector competence studies
Source: PLoS Negl Trop Dis. 2023 Aug 31;17(8):e0011591. doi: 10.1371/journal.pntd.0011591 (PMC10499269; doi:10.1371/journal.pntd.0011591)
Supplement: Attachment — Submitted filename: Revision2responses.docx [file pntd.0011591.s005.docx]

General Response: Again, we would like to thank all the reviewers for their efforts, in particular the request to check Table 5 yet again, and the very helpful and specific grammatical corrections. For the latter, it illustrates the benefits of independent copy editing and is always amazing and frustrating the obvious grammatical errors that are missed by authors who have read the manuscript way too many times. For Table 5, we have created an excel spreadsheet with the raw metadata extracted from each manuscript and calculated rates with formulas. This will now be included as supplemental information and should have been our approach from the beginning. This at a minimum eliminates the inversion errors (we switched some stepwise and cumulative rates when putting in the table), but a few articles posed significant problems in extracting the metadata (see responses below). These cases have footnotes in the table legend describing some of the assumptions we had to make, but highlight many of the problems in data reporting we describe in the manuscript. All responses are provided in blue.

Revision #2 Responses.

Reviewer's Responses to Questions

-Are the objectives of the study clearly articulated with a clear testable hypothesis stated?</br> -Is the study design appropriate to address the stated objectives?</br> -Is the population clearly described and appropriate for the hypothesis being tested?</br> -Is the sample size sufficient to ensure adequate power to address the hypothesis being tested?</br> -Were correct statistical analysis used to support conclusions?</br> -Are there concerns about ethical or regulatory requirements being met?</br></br>

Reviewer #1: See general comments.

Reviewer #2: Accept

Reviewer #3: Yes,Yes,Yes,N/A,N/A,No

--------------------

<b>Results</b></br></br>

-Does the analysis presented match the analysis plan?</br> -Are the results clearly and completely presented?</br> -Are the figures (Tables, Images) of sufficient quality for clarity?</br></br>

Reviewer #1: See general comments.

Reviewer #2: Accept

Reviewer #3: Yes,Yes,Yes

<b>Conclusions</b></br></br>

-Are the conclusions supported by the data presented?</br> -Are the limitations of analysis clearly described?</br> -Do the authors discuss how these data can be helpful to advance our understanding of the topic under study?</br> -Is public health relevance addressed?</br></br>

Reviewer #1: There are some issues. Please see general comments.

Reviewer #2: Accept

Reviewer #3: Yes,Yes,Yes,Yes

<b>Editorial and Data Presentation Modifications?</b></br><br/> Use this section for editorial suggestions as well as relatively minor modifications of existing data that would enhance clarity. If the only modifications needed are minor and/or editorial, you may wish to recommend “Minor Revision” or “Accept”.

Reviewer #1: There were numerous minor grammatical issues. I pointed many of these out in the general comments.

Reviewer #2: Accept

Reviewer #3: (No Response)

<b>Summary and General Comments</b></br></br> Use this section to provide overall comments, discuss strengths/weaknesses of the study, novelty, significance, general execution and scholarship. You may also include additional comments for the author, including concerns about dual publication, research ethics, or publication ethics. If requesting major revision, please articulate the new experiments that are needed.

Reviewer #1: General Comment: This version is much improved. However, I am still a little concerned about how transmission rate is used in this paper. The use in Table 5 is fine but its use in the text can be misleading (see comment 8 below). Also, there were numerous minor editorial errors (multiple uses of a numeral, at random, for numbers less than 10). To me, this illustrates some carelessness. More importantly, many of the rates given in Table 5 are wrong (see comment 5 below).

Response: The grammatical rules provided by Reviewer #1 were very helpful, and we googled the topic and our interpretation of measurements was clearly too wide. Fortunately, Reviewer #1 pointed out our errors and we have made those modifications. We will comply with PLOS recommendations. As for Table 5, we have created an excel spreadsheet (see response below), to reduce some of the more careless errors and appreciate Reviewer #1’s ability to spot these errors, it is much appreciated and we hope the reviewer can sympathize with the author’s fatigue, after multiple checks of this Table last round.

Specific Comments:

1. Line 124: Vector competence studies generally do NOT include “inoculation” or ‘intrathoracic inoculation,” but may use that technique to examine either midgut escape or salivary gland barriers. Inoculated mosquitoes cannot be used to determine a transmission rate as this technique bypasses two critical barriers, midgut escape and salivary gland barriers.

Response: I cited this text from Wu et al., but happy to remove both words, since the focus of our article is “transmission” and less about establishing where barriers to transmission our occurring, but I would respectable disagree they do not represent a subset of vector competence studies. We discuss the issue you brought up in detail to describe the value of SDR versus CDR. (see Lines 124-126).

To provide further support to the key message of vector competence studies designed to implicate a vector we have added the following sentence to line 140

“The cumulative transmission rate alone implicates a species as a vector.”

2. Table 1, Guo reference: As they did not check for actual, infectious virus, isn’t it much more likely that they were detecting lingering small pieces of noninfectious RNA, rather than a transient infection, which as you indicate would be totally new and unexplained paradigm?

Response: You are completely correct in your assessment of the PCR data, but they also did experiments putting presumably infected mosquitoes in cages with neonatal mice and 8 of 9 of the mice had viral RNA in their brain, with respectable quantities of RNA copies/ml. Seems like if this data is to be believed you would need to call this transmission, but I would be very interested in knowing the caveats of interpreting this mouse data. Can one explain the presence of viral RNA in the brain of those mice without transmission having occurred, if not how. The traditional VC experiment data is not consistent with transmission in our opinion and personally I have some concerns about the mice data, but short of fraud I don’t have an explanation. I wish this had been strongly challenged at the time, because this manuscript remains the most cited on the topic and the most difficult to explain away.

3. Lines375-383: I added up the numbers for the various locations, 16, 10, 5, 4, 4, 2, 1 (not identified), and 2 (multiple continents), and the total was 44. Where was the missing study done? Based on Table 2, shouldn’t the 16 for the Americas be 17?

Response: Another excellent catch and much appreciated. Line 375 now states 38% (17 articles). Embarrassed that this among other items were missed.

4. Lines 388-389: Why is Armigeres not listed on line 388? Li et al. did test the vector competence of this species for ZIKV.

Response: As Reviewer #1 had originally requested that we state that studies on Armigeres also tested the hypothesis that infection could occur via exposure to urine in human waste pools, we highlight this paper in the following sentence. This was our rational for not listing we did not list it line 387. For clarity, we added Finally, at the beginning of the sentence and changed “susceptibility” to vector competence. (see lines 387-388)

5. Table 5: Much better, but there are still some problems.

a. For Ae. luteocephalus, how is it possible for the CDR to be 42% if the SDR was only 37%? By definition, the SDR has to be greater than or equal to the CDR and (c.) For Aedes vitalis, how can the CDR be 17% if only 14% of the mosquitoes were infected?

Response: Reviewer #1 was correct for the need to check Table 5, there were a number of unforced errors and the Table is updated. For these two species however, both from Diagne et al. we speculate that there were some errors in the original manuscript, and raw numbers were presented in the text and not in tabular format. For some parameters absolute numbers were back calculated from percentages provided. For example, for Ae. leutocephalus and Ae. vittatus, numbers were provided in text in parallel. Text clearly states 60 mosquitoes were exposed, 45 were infected and 42% of the 45 infected mosquitoes had disseminated infections (18.9 mosquitoes saying 19). Text says 50% of 27 saliva’s tested positive for this species. So, we think there is an error somewhere here in the original article. I assumed it should be 50% of 19 and used that to calculate the CTR (10/60-17%) which has a footnote. This is not the only example, where extraction of the metadata was not straight forward and it was not always clear how the authors did their experiments.

b. For Ae. vigilax, how is it possible for the CDR to be 27% if the SDR was only 10%?

Response: corrected SDR=47% and CDR=27%, also identified transcription error for Ae. procax which has been corrected.

d. I am a little confused about the calculations for Ae. japonicus. If the IR was 87%, and the CDR was 19%, should the SDR have been 22%, not 27%. Alternatively, if the SDR was 27%, shouldn’t the CDR have been 22%. There is the same problem with the TRs. If the SDR was 27 and the STR was 57%, then the CTR should have been 15%, not 11%. However, if the SDR was 22% (see above) and the STR was 57%, then the CTR should have been 12.5% (note, the 1% difference might be due to the decimal points not shown in the original data.

Response: corrected, the example cited above 120 mosquitoes were exposed, 104 became infected, 23 had disseminated infections, and 13 had virus in saliva for IR=87% SDR=22%, CDR =19%, STR=57% and CTR 11%. I’ve checked the calculations, and there was an error for SDR, but CTR remains 11% by my calculations.

e. As illustrated by the above four examples, there are some errors in the calculations of these rates. Please go back and confirm all of the rates as I found several others that are also wrong.

Response: We appreciate Reviewer #1’s diligence, because indeed there were several error, and we will now include a spreadsheet with the extracted values and calculations in supplemental information.

f. The importance of clarifying the transmission rate definitions is illustrated by Sa. cyaneus. If the paper defined TR as the number transmitting/number tested, then the TR was 1%. However, if they used the inappropriate definition of number transmitting/number disseminated, it was 100%. In the abstract of the paper, it would have said, the TR for Sa. cyaneus was 1% (or 100%) depending on the definition used in the paper. Unfortunately, most people only read the abstract to get this information.

Response: Below is he relevant component of the abstract from that paper, and you can see these authors were quite transparent and chose their words carefully, however, we had some concerns that 1 positive mosquito in all body compartments was a bit strange.

Of 69 Sa. cyaneus that fed, ZIKV was detected in only one, in all body compartments, at 21 dpf. In contrast, at 14 dpf 100% of 20 Ae. aegypti that fed on mice at 2 dpi were infected and 70% had virus in saliva. These data demonstrate that Sa. cyaneus is a competent vector for ZIKV, albeit much less competent than Ae. aegypti.

6. Lines 549-551: As illustrated by the example of Sa. cyaneus in 5(f) above. The definition of transmission rate is critical. If you used the STR, the transmission rate would be 100%, i.e., more efficient that Ae. aegypti, but if you used the CTR, then it would be 1% and extremely inefficient. You need to explain which you are using, and if using the STR, then some extremely inefficient vectors would be classified as extremely efficient, and this is not only misleading, but public health people might focus their control on an extremely inefficient species and ignore more important vectors, resulting n more disease.

Response: This statement was correct because the Ae. aegypti strains tested did not transmit ZIKV, however your point is well taken and we have modified that sentence to say the following (Lines 550-552)

“The only forest species with both laboratory evidence incriminating them as a ZIKV vectors were *Ae. vittatus* and *Ae. luteocephalus*, both able transmit virus with CDR of 1 and 17%, respectively.”

7. Lines 568: Again, you need to be clear as to which definition of dissemination you are using for the various studies. Species with a relatively low infection rate may appear to have a high dissemination rate, making them look much more efficient.

Response: We have modified Lines 567-570 to say the following:

“In contrast, a later study showed two populations of *Ae. polynesiensis* from French Polynesia and Wallis and Futuna had high infection rates (71%) that were less variable than *Ae. aegypti* populations from the same region and showed high dissemination rates (SDR=45%, CDR=39% and low transmission rates (STR=3%, CDR=1%) (57).”

8. Lines 574-576: This really illustrates the problem. If only 34% of the Ae. notoscriptus became infected (and only 3% had a disseminated infection), how could they have a 42% transmission rate when only about 1% of the virus-exposed mosquitoes actually transmitted ZIKV? Obviously, this species is a very inefficient vector of ZIKV. However, someone reading this sentence would think that it was an efficient vector. This is very misleading.

Response: Table 5 has been updated. The Duchemin et al. paper this is derived from does not provide raw numbers and it is almost impossible to determine from the text if the percentages the report are cumulative or stepwise – the text implies stepwise but only cumulative make any sense. We provide our rational in Table S2 and Table 5 footnotes. Additionally, dissemination was based on positive carcasses also flawed methodology. In our previous calculations we assumed that stepwise rates were being reported, but upon reexamination this can not be possible (numbers just do not add up).

Now lines 575-577 have been modified to say.

“In contrast, a later study (48) found some transmission (<1%), but lower infection and dissemination rates than the previous study for both *Ae. notoscriptus* and *Ae. camptorhynchus*.”

9. Lines 632-634: You might want to add that noninfectious pieces of RNA have been shown to persist for long periods of time in a mosquito, and thus, this study detected these noninfectious pieces of RNA, but the amount of these noninfectious pieces would decline with time providing the results reported.

Response: we have changed the text to say the following (Line 635-636)

“a result consistent with the observed detection of decreasing levels of noninfectious pieces of RNA rather than infectious virus”.

10. Lines 810-814: This is important.

Response: We hope this paragraph clearly makes the point that cumulative rates are what should be considered when accessing if a species is a potential vector or not, addressing this important concern by Reviewer #1.

11. Lines 817-820: Yes. If someone reads the paper, they can understand how the authors defined a TR. However, many of these papers simply state in the abstract that the TR for species X was 50% and most readers do not read the paper. However, if they had used a stepwise rate and only two of the 100 mosquitoes tested had a disseminated infection, and one of these transmitted, then instead of being an efficient vector, as indicated in the abstract, it would be a very inefficient vector. As indicated in the paragraph, this could have serious public health liabilities. We need to be consistent in our definition of a transmission rate, and if the purpose is to identify potential vectors, then it needs to be the number transmitting/number tested.

Response: We have added the following sentence at the end of the paragraph of concern (see lines 821-822), to definitively state that those in the field to agree to adopt this standardized terminology. Obviously, enforcing it would be at the level of editorial/review process.

“Moving forward, our recommendation is the universal adoption of the terminology for cumulative and stepwise rates as we have presented in Table 5.”

12. Lines 835-839: Thanks for pointing this out.

Response: At Reviewer #1’s suggestion.

13. Lines 855-858: I’ve played with this quite a bit. I have often found that a single mosquito bite on the tip of a mouse’s tail usually contains >1,000 PFU of virus, but a saliva sample rarely contained mor than 100 PFU, and a mosquito that had transmitted to a rodent often contained no virus in its saliva sample. While it is easier and less costly to simply collect saliva and test it, it tends to underestimate the actual TR. This is consistent with what you are reporting.

Response: In this group of papers, most just tested saliva, but methods of amplification, for example we would inoculate clean Ae. aegypti mosquitoes. Interestingly, if one reviews manuscripts for Ae. aegypti and Ae. albopictus this becomes a more important issue. But we have made no change to this paragraph.

Minor Comments:

14. Line 98: “VC” needs to be established in the main body text.

Response: See line 95 where we had done this.

15. Line 119: “vector capacity…” should be “vectorial capacity…” Done

16. Line 148: What is “transmission efficiency?”

Response: Transmission efficiency is a term that has permeated the literature, but we now say (Lines 146-147)

“and parameters assessed (both stepwise and cumulative infection, dissemination, transmission rates) are all critical for assessment of VC”

17. Line 163: This does not read well. Should “where” be added so that it is now “a period where VC studies…?” done

18. Line 319: Why is “Days” post exposure capitalized, while “days” post infection is not? done

19. Line 319: “contain…” should be “containing…” done

20. Line 334: Why establish “MS” if it is not used again? This term in used in Table 1 (final column)

21. Line 335: Why establish “TS” if it is not used again? This term in used in Table 1 (final column)

22. Line 388: I don’t think that you need both “genus” and “genera” in this sentence. That is repetitive. done

23. Line 400: “4 articles…” should be “four articles …” done

24. Line 413: “using well described the viral strains…” should be “using well described viral strains…” done

25. Line 469: Why establish “DIR” or “YR(D)” if they are not used again?

Response: Because these abbreviations are used in the literature, which I hope will continue to clarify the problem with definitions some are recognized by their abbreviations.

26. Line 493: Why report a titer to a hundredth of a log? This is not accurate. I don’t care if that was reported in the paper cited, it should be 6.8 logs. done

27. Line 501: “ten mosquitoes…” should be “10 mosquitoes…” done

28. Line 663: “these kind studies…” should be “these kinds of studies…” done

29. Line 702: Why establish “YF” if it is not used again? See Reviewer #3 comment below

L155: yellow fever is first mentioned here, then again at L420, then at L702 (where it is abbreviated YF) and L745-746 (“yellow fever” again). The abbreviation may come at L155 and YF be used later for clarity and consistency. done

30. Line 760: “1 or 2 local…” should be “one or two local…” done

31. Line 929: Shouldn’t “rates primary vectors…” be “rates than primary vectors…? done

32. References

a #16 (Cao-Lormeau): Shouldn’t “Polynesia” be capitalized? Done

b. #18: Why is “Transactions of The Royal Society of Tropical Medicine and Hygiene” written out. Check others. done

c. #37: Why is “chikungunya” capitalized? done

Reviewer #2: In the revision of the review “Secondary vectors of Zika Virus, a systematic review of laboratory vector competence studies” Bisia and colleagues have addressed and/or justified their rationale for comments and concerns expressed by myself and the other reviewers. As such, I support the publication of the manuscript in its current form.

Response: Thanks

Reviewer #3: Following my initial review and that of the other two anonymous reviewers, the authors have substantially improved their manuscript and I can only commend them for going through what must have been some very tedious modifications and proof-reading.

Response: Reviewer #3 is correct, but it was warranted and indeed led to a much better manuscript, this is a good example of how the review process should function.

My main criticism at first submission was the long delay between the end date for including papers in this systematic review and timing for submission (more than a year). This resulted, among other things, in reviewing that paper after I had myself published what the authors were calling for as “future efforts” in the discussion. In an effort for transparency, I decided it was only fair that I disclosed my name during the review process, and that hopefully it did not make me sound like an author wanting his word credited. I appreciate that for any systematic review, and end date must be set and respected. It is unfortunate that publication of this paper will happen more than a year and a half later, though we can all agree that “publishing science takes time” and sometimes delays out of authors’ control contribute to lengthy publication process.

Response: No worries at all, the paper mentioned illustrates are point exactly, and it fit well into our discussion. As an aside, we wrote a systematic review back in 2017 that focused on all vectors only to be scooped by Epeloin et al. I (senior author) wanted to rerun the search because many new manuscripts had emerged, but it was not easy to coordinate. Responding to these reviews illustrates how much time and effort a good systematic review requires, we had run out of steam a bit, but I now this will be a helpful manuscript to the field.

Among the strongly positive points now made by the authors is the call for harmonized reporting standards (e.g. clearly defining stepwise/cumulative rates), a major point raised by anonymous Reviewer #1. I can only hope that future VC work will build upon that terminology. The QAS, though remaining of high relevance, also feels better now that it is introduced as “guidelines” towards standardization rather than a rewarding metric.

Response: Thanks

I now only have minor comments that, once addressed, will make that paper acceptable for publication. Upon reading that R1 revision, I consider my major comments addressed and can recommend this article for publication PLoS Neglected Tropical Diseases pending corrections for remaining typos and few syntactical errors.

Minor comments

L155: yellow fever is first mentioned here, then again at L420, then at L702 (where it is abbreviated YF) and L745-746 (“yellow fever” again). The abbreviation may come at L155 and YF be used later for clarity and consistency. done

L295: “considers” should probably not have an “s” here. done

L539+: consider mentioning somewhere that the now harmonized “NT” stands for “Not tested”. done

L725: “accessing” should probably be “assessing”. This was actually a minor suggestion from my first review and marked as “done” by the authors, could you double-check that other small typoes highlighted earlier were not corrected then wrongly rejected/reverted ?

Response: we did a search for access and found numerous errors, thanks for the suggestion.

L762: Missing an “s” at “provides”. done

L801: accessing/assessing, again. Now assessing

L851-853: the sentence does not make sense as is though I think understand what the authors meant. Consider inverting the beginning so that it’s clear that these constraints dictate what can and can’t be reasonably expected from low/middle-income countries in terms of lab capacity and experiments.

Response: Sentence rewritten as suggested to ready (Lines 853-856)

“The ability to implement more realistic and appropriate study designs for VC studies in low- and middle-income countries where ZIKV and other arboviruses are endemic may be limited due to financial and logistical restraints.”

L889: Shouldn’t “geographically” be “geographical”? yes and done

--------------------

PLOS authors have the option to publish the peer review history of their article (<a href="<https://journals.plos.org/plosntds/s/editorial-and-peer-review-process#loc-peer-review-history>" target="_blank">what does this mean?</a>). If published, this will include your full peer review and any attached files.<br><br>

If you choose “no”, your identity will remain anonymous but your review may still be made public.<br><br>

<b>Do you want your identity to be public for this peer review?</b> For information about this choice, including consent withdrawal, please see our <a href="<https://www.plos.org/privacy-policy>" target="_blank">Privacy Policy</a>.

Reviewer #1: No

Reviewer #2: No

Reviewer #3: Yes: Thomas Obadia

Figure Files:

While revising your submission, please upload your figure files to the Preflight Analysis and Conversion Engine (PACE) digital diagnostic tool, <https://pacev2.apexcovantage.com>. PACE helps ensure that figures meet PLOS requirements. To use PACE, you must first register as a user. Then, login and navigate to the UPLOAD tab, where you will find detailed instructions on how to use the tool. If you encounter any issues or have any questions when using PACE, please email us at [figures@plos.org](mailto:figures@plos.org).

Data Requirements:

Please note that, as a condition of publication, PLOS' data policy requires that you make available all data used to draw the conclusions outlined in your manuscript. Data must be deposited in an appropriate repository, included within the body of the manuscript, or uploaded as supporting information. This includes all numerical values that were used to generate graphs, histograms etc.. For an example see here: <http://www.plosbiology.org/article/info%3Adoi%2F10.1371%2Fjournal.pbio.1001908#s5>.

References

Done
