# Supplementary material for: Secondary vectors of Zika Virus, a systematic review of laboratory vector competence studies
Source: PLoS Negl Trop Dis. 2023 Aug 31;17(8):e0011591. doi: 10.1371/journal.pntd.0011591 (PMC10499269; doi:10.1371/journal.pntd.0011591)
Supplement: Attachment — Submitted filename: reviewerResponsesR3.docx [file pntd.0011591.s006.docx]

Reviewer's Responses to Questions

Reviewer #1: General Comment: This version is much improved. However, the are still a few issues that need to be corrected. Please see my comments below.

Specific Comments:

1. Line 406: “ten generations…” should be “10 generations…” done

2. Table 5

a. What does “Raw data presented in” at the end of the table heading mean?

Changed to “Raw data presented in Table S2.”

b. For Ae. notoscriptus, you report IR- 29%, SDR-21%, CDR-10%. However, if 29% are infected, and 21% of those have a disseminated infection, then only 6% of the total would be infected. On the other hand, if the CDR was 10% and 29% were infected, the SDR would be 34%

Please see Table S2, but again thanks to your eye I can see that the table got misaligned, so we had two studies that were presented separately. Numbers have been updated in Table 5.

c. The results for Ae. vigilax IR- 34%, SDR-47%, CDR-27% ref43 and Ae. procax IR- 57%, SDR-50%, CDR-17% ref 43 seem to be partially crossed. The SDR-50% and CDR-17% reported for Ae. procax work for Ae. vigilax, and the SDR-47% and CDR-27% reported for Ae. vigilax work for Ae. procax. Alternatively, the two infection rates were switched.

Also part of the misalignment problem.

3. Line 550: What does, “with both laboratory evidence” mean? Eliminated “both”

4. Line 558: Yes, it was only one mosquito that became infected and transmitted ZIKV. However, the sentence gives no indication of the sample size, so if they had only tested that one Sa. cyaneus, it would appear to be a very efficient vector. The authors might want to modify the sentence to, “…had one mosquito out of 60 tested with…

Added “out of 69 tested".

5. Line 583: Why use the word “Important” for those two species, particularly as they were refractory. Delete “Important.” Deleted.

6. Line 674: I agree that that data sets should be provided. However, how are the authors going to guarantee that they “will be required.” Shouldn’t this be, “New studies should be required to do so…”

“will” changed to “should” NOTE: many journals are now requiring this.

7. Line 824: Shouldn’t “Both these definitions…” be “Both of these definitions…?

Added “of”

8. Lines 852-858: You might also want to cite Turell MJ. Reduced Rift Valley fever virus infection rates in mosquitoes associated with pledget feedings. Am J Trop Med Hyg. 1988 Dec;39(6):597-602[1] that clearly showed that when feeding mosquitoes (both Culex and Aedes) on blood from a viremic hamster or on the hamster itself, the infection rate in the mosquitoes was significantly higher if they had fed on the hamster although the amount of virus ingested was identical.

Added suggested reference.

9. Line 949: Thanks

Well deserved.

Reviewer #2: In the revision of the review “Secondary vectors of Zika Virus, a systematic review of laboratory vector competence studies” Bisia and colleagues have further addressed concerns, in particular in regards to Table 5, and the definitional consideration between stepwise vs cumulative dissemination/transmission rate. In revising, the authors have strengthened an already phenomenal manuscript. Given that, I would wholeheartedly endorse the acceptance and publication of this work.

Thank you kindly!

Reviewer #3: My minor concerns mentioned after Revision 1 have been addressed.

Thanks

The authors now provide Table 5, which raised numerous concerns (most of these detected by the very thorough anonymous Reviewer #2), has now been derived from an Excel spreadsheet with automated calculations. This makes sure that IR >= CDR >= CTR. The formulas have not been applied to all rows, presumably to avoir '#ERROR' values due to some divisions by zero, and instead value was forced to zero in these cases. I am honestly not sure which is best since 0/0 is also not equal to 0, though readership will eventually be using "0" for any dissemination/transmission rate if infection was completely unsuccessful. So, this is fine by me.

Good point, but thanks.

There are rows where formulas do not reference existing cells:

- Some with hard-coded values that were derived based on extrapoliting percentages in original articles; this is fine by me

- Two have hard-coded mathematical operations, this is slightly worse because one has to make sure the numbers do match those from columns B, C, D and E. This applies to row 20 (I20 and J20)

Copied the formulas over.

Since this supplementary file is used as a basis for generating Table 5, it would be good that at least these two hard-coded mathematical operations are corrected to reference the corresponding denominators from columns B and D. The results are, nonetheless, valid.

Made corrections

I am fine with proceeding for publication (or asking the authors for that very minor modification to Table S2 and then only undergo editorial validation).

Done

--------------------
